# Genome-Wide Identification of the GRAS Transcription Factor Family in Sweet Orange and the Regulation of Salt Stress-Enhanced Plant Salt Tolerance in Sweet Orange by *CsGRAS15* and *CsGRAS27*

**DOI:** 10.3390/biom15070946

**Published:** 2025-06-29

**Authors:** Hailin Ren, Rong Xu, Jie Wang, Qian Zhang, Lili Nie, Li Zhang, Xianyan Zhou, Xiaozhen Liu, Hanyao Zhang

**Affiliations:** 1 Forestry College, Southwest Forestry University, Kunming 650224, China; 2113676876@swfu.edu.cn (H.R.); wangjie1201@swfu.edu.cn (J.W.); 871970420@swfu.edu.cn (Q.Z.); nielily@swfu.edu.cn (L.N.); 2 School of Chemical, Biological and Environmental, Yuxi Normal University, Yuxi 653100, China; xurong@yxnu.edu.cn; 3 Yunnan Agricultural Technology Extension Station, Kunming 650000, China; zhangli15669293@gmail.com; 4 Institute of Tropical Subtropical Economic Crops, Yunnan Academy of Agricultural Sciences, Baoshan 678000, China; zhouxianyanswfu@gmail.com

**Keywords:** sweet orange, salt-responsive, GRAS transcription factor, signaling pathway

## Abstract

Background: GRAS transcription factors are crucial for plant development and stress responses but remain poorly characterized in citrus. Soil salinization increasingly threatens sweet orange (*Citrus sinensis*) yield. Identifying salt-responsive *GRAS* genes could reveal key tolerance determinants for breeding resistant cultivars. Methods: We systematically identified and analyzed sweet orange GRAS transcription factors using bioinformatics. Results: Forty-three *CsGRAS* genes were identified, phylogenetically classified into ten subfamilies, and found to be structurally conserved. A promoter analysis revealed a high prevalence (58.78%) of hormone- and stress-responsive cis-elements. These genes reside on nine chromosomes, with segmental duplication being the primary evolutionary driver (eight duplicated pairs). Functional enrichment implicated hormone signaling pathways in regulating growth under stress. Transcriptome profiling identified 42 differentially expressed *CsGRAS* genes (19 upregulated and 23 downregulated) under salt stress. qRT-PCR validated the expression patterns of selected genes (e.g., *CsGRAS15* and *CsGRAS27*). Notably, DELLA subfamily members *CsGRAS15* and *CsGRAS27*, key negative regulators in gibberellin (GA) signaling, were differentially expressed. Modulating these DELLA proteins presents a promising strategy to enhance sweet orange salt tolerance by mitigating GA-mediated growth inhibition during stress. Conclusion: This study identifies salt-responsive *CsGRAS* genes, highlighting *CsGRAS15* and *CsGRAS27* as potential targets for improving salt tolerance in citrus.

## 1. Introduction

Sweet orange (*Citrus sinensis* (L.) Osbeck) is a tree species of the genus Citrus in the family Rutaceae (corrected from Brassicaceae) with many unique characteristics and a wide range of applications [1]. Sweet oranges can be consumed fresh or processed into juice, canned foods, or other products, such as orange peel. Their fruit is rich in nutrients, such as vitamin C and organic acids, and has a high nutritional value [2]. Data show that the global distribution of sweet oranges exhibits significant geographical patterns, with Asia serving as the primary producing region, accounting for more than half of the global production share (>50%), highlighting its extensive cultivation and importance in this region [3]. However, in recent years, the growing severity of soil salinization has adversely affected the growth, development, yield, and fruit quality of sweet oranges [4]. Therefore, research on salt-tolerant genes and germplasm resources of sweet oranges has become both critical and urgent.

The high osmotic potential of saline soils lowers soil water potential, impeding root water uptake and inducing “physiological drought.” This process rapidly suppresses cell expansion and leaf growth within minutes to hours [5]. Inadequate water supply increases xylem water column tension, leading to air pocket (embolism) formation and reduced hydraulic conductance [6]. Furthermore, excess Na^+^ displaces Ca^2+^ from cell membranes, destabilizing them, and competitively inhibits K^+^ uptake, resulting in cellular Na^+^/K^+^ imbalance [7]. Finally, salt stress triggers excessive accumulation of reactive oxygen species (ROS) in chloroplasts and mitochondria. Superoxide anions (O_2_^−^) and hydrogen peroxide (H_2_O_2_) attack membrane lipids and proteins, causing lipid peroxidation and chlorophyll degradation [8]. Differences in antioxidant capacity constitute a key factor underlying variation in plant salt tolerance. Collectively, salt stress disrupts plant physiological homeostasis through the combined effects of osmotic stress, ionic toxicity, and oxidative damage, ultimately leading to growth inhibition and plant mortality.

Transcription factors are a class of proteins with specific recognition and binding capabilities that can interact highly specifically with cis-acting elements in gene sequences. Through these interactions, transcription factors can act as regulators, either by inhibiting or activating the transcription process of genes, thus finely regulating the gene expression level. Transcription factors are also known as transacting factors; this reversal has led to e-acting properties in transcriptional regulation [9]. In recent years, with the development of science and technology, whole-genome sequencing of soybean (*Glycine max* (L.) Merr.), Arabidopsis (*Arabidopsis thaliana* (L.) Heynh.), and rice (*Oryza sativa* L.) [10] has been completed. A series of transcription factors related to abiotic stresses have been successively identified in these plants. A series of transcription factors related to abiotic stresses, such as SAP2/ERF [11], bZIP [12]), WRKY [13], and GRAS [14], have been identified from these plants. GRASs constitute a family of plant-specific transcription factors that play vital roles in regulating growth and developmental processes and responding to adverse stresses in plants.

The GRAS transcription factor family includes three genes that were identified in plants and exhibit crucial functions: gibberellic acid insensitive (GAI), repressor of GA1 (RGA), and scarecrow (SCR) [15]. This nomenclature reflects their key role in the signaling pathway of the plant hormone gibberellin (GA) and underscores their wide-ranging impact on plant development regulation. Although GRAS proteins may share structural similarities with members of the Rossmann fold methyltransferase superfamily [16], they are plant-specific transcription factors, defined primarily by their nuclear localization, DNA-binding ability, and transcriptional activation properties [17]. The GRAS transcription factor family is notably characterized by polypeptide chain lengths typically varying between 360 and 850 amino acids. The protein structure of family members can be divided into two main parts: a highly variable N-terminal domain and a highly conserved C-terminal domain [18]. They contain five specific motifs—LHRI, VHIID, LHRII, PFYRE, and SAW—which form the GRAS domains unique to this family [19]. The N-terminus harbors two conserved structures of DELLA and TVHYNP proteins, both associated with GA signaling [20].

With the development of genetics and genomics, significant progress has been made in the study of the GRAS transcription factor family. Genome-wide identification and analysis of members of this family have been completed in a wide range of plants, including but not limited to the model plant *A. thaliana* [21], the vital food crop rice [22], the forest resources poplar (*Populus* L.) [23], pine (*Pinus* L.) [24], and grapevine (*Vitis vinifera* L.) [25], as well as those widely used in the agricultural production of soybean [26]. Members of the GRAS transcription factor family in the whole genome of sweet oranges were analyzed in this study. Member identification, physicochemical property analysis, phylogenetic tree construction to trace their evolutionary history, the analysis of gene structure, structural domains and conserved motifs, and the identification of cis-acting regulatory elements to reveal their transcriptional regulatory mechanisms were performed; chromosomal localization, gene duplication, and covariance analysis were conducted; and the prediction of their involvement in biological processes via GO and KEGG enrichment analysis was performed. Importantly, this study focused on the expression patterns of GRAS transcription factors in response to salt stress conditions in sweet oranges, aiming to reveal the potential roles of these transcription factors in plant stress tolerance, especially salt stress tolerance. Through the above comprehensive analyses, the present study provides a solid theoretical foundation for the evolutionary history and biological functions of the sweet orange GRAS transcription factor family.

## 2. Materials and Methods

### 2.1. Access to Data and Materials

Sweet orange genome data (DVS_A1.0) were downloaded from the Citrus Pangenome to the Breeding Database (http://citrus.hzau.edu.cn/download.php (accessed on 28 June 2024)) and the National Center for Biotechnology Information (NCBI) [27,28]. Sequence information on the conserved structural domain of the GRAS transcription factor family (ID: PF03514) was downloaded from the Pfam website (http://pfam.xfam.org/ (accessed on 28 June 2024)). The protein sequences and genomic data of 33 Arabidopsis GRAS transcription factor families were downloaded from the Arabidopsis genome website TAIR (https://www.Arabidopsis.org/ (accessed on 29 June 2024)) [21].

### 2.2. Genome-Wide Identification and Preliminary Physicochemical Characterization of the GRAS Transcription Factor Family in Sweet Orange (Citrus sinensis)

For the results of the downloaded data checked from the above website, first, the downloaded HMM mapping of *GRAS* structural domains was used for HMMER (v3.3.2) to identify potential *GRAS* genes from the sweet orange genome and screen the sequences of the family members with an E-value < 1 × 10^−5^ (statistical threshold for homology significance) [29]. Secondly, domain detection was performed using BLAST (v2.13.0) software and the Pfam *GRAS* model (PF03514) with the default aggregation threshold. The protein sequences of the Arabidopsis GRAS transcription factor family were used as a reference and compared with the protein data of the sweet orange genome to screen for protein sequences with high homology (E-value < 1 × 10^−5^) to the Arabidopsis GRAS transcription factor family [21,30]. Finally, the obtained sequence numbers were merged, and the corresponding sequences from the sweet orange genome protein data were extracted using the TBtools (v1.113) software. The extracted sequences were then saved in FASTA format [31]. At the same time, the Conserved Domain Database (CDD) of NCBI (https://www.ncbi.nlm.nih.gov/Structure/cdd/wrpsb.cgi (accessed on 10 July 2024)) and PFAM (http://pfam.xfam.org/search#tabview=tab1 (accessed on 11 July 2024)) online tools were used to verify the existence of GRAS structural domains, and based on the validation results of CDD and PFAM, the sequences from Sweet Orange GRAS_Proteins were rapidly removed to remove sequences that were absent or had incomplete GRAS structural domains. The filtered sequences are saved as a new fasta file, e.g., Sweet Orange GRAS_TFs.fasta. Having completed the identification of the sweet orange GRAS transcription factors, the next step in this study is to rename them based on their position on the chromosome, first by collating the chromosomal position information (to ensure that the position information for each GRAS transcription factor on the chromosome is available). Second, assign sequential numbers: assign a unique sequential number to each GRAS transcription factor based on the chromosome position or any chosen sequential criterion (if positional information is unavailable or too complex). Third, renaming: new names were created for each GRAS transcription factor in the format *CsGRAS1*-*CsGRAS43*. Physicochemical property analysis of proteins can be performed via ExPASy’s ProtParam tool (https://www.expasy.org/ (accessed on 15 July 2024)), an online bioinformatics resource that predicts essential protein characteristics. This platform enables comprehensive analysis including molecular weight (MW) determination, protein length calculation, isoelectric point (pI) prediction, amino acid composition quantification, and the evaluation of stability parameters, such as the instability index and aliphatic index [32]. The final prediction of the subcellular localization of the GRAS protein sequence can be performed via BUSCA (http://busca.biocomp.unibo.it/ (accessed on 15 July 2024)).

### 2.3. Sequence Similarity and Phylogenetic Analysis

In this study, Clustal Omega (v1.2.4) (https://www.ebi.ac.uk/jdispatcher/msa/clustalo (accessed on 17 July 2024)) was used to analyze the homology among sweet orange *GRAS* gene families by performing multiple sequence comparisons and calculating sequence similarity. TBtools was used to map the correlations among family members based on the sequence similarity heatmap. To delve deeper into the evolutionary relationship of the GRAS transcription factor family between the sweet orange (*C. sinensis*) and the model plant Arabidopsis (*A. thaliana*), a series of bioinformatic analysis steps were taken. GRAS protein sequences were initially systematically identified and retrieved from sweet orange (*C. sinensis*) and Arabidopsis (*A. thaliana*), followed by their integration for comprehensive downstream analyses. In the sequence comparison stage, alignment was performed using MEGA 11.0 (--auto mode) [33] and 1000 bootstrap repetitions were performed to identify conserved regions and variant sites between sequences. Immediately after that, the preliminary comparison results were trimmed via TBtools software to remove uncertain regions and redundant information in the comparison results to ensure the accuracy and reliability of the subsequent analyses. To construct the phylogenetic tree, we selected the best-fitting model in IQtree (ModelFinder, BIC criterion) [32] + 1000 ultra-fast bootstrap method. By combining MEGA 11.0 software and IQtree, we constructed the phylogenetic tree of the GRAS transcription factor family in sweet orange and Arabidopsis. The phylogenetic tree reveals the evolutionary distance between these transcription factors, as well as their potential evolutionary relationships and taxonomic groups. To more intuitively display the structure and information of the phylogenetic tree, nodes with bootstrap support rates below 70% were collapsed (consensus threshold). This study selected the online software Evolview (http://www.evolgenius.info/evolview/#/treeview (accessed on 20 July 2024)) to visualize the evolutionary tree. Finally, 43 sweet orange GRAS proteins were grouped according to published Arabidopsis GRAS protein grouping criteria [34].

### 2.4. Chromosomal Localization, Duplicate, and Covariance Analysis

Chromosome location information can be obtained from the annotation information and the whole-genome protein sequence of sweet orange. The chromosomal location information in the target gene’s GFF annotation file was extracted and visualized through the ‘Gene Location Visualize from GFF’ function integrated into TBtools software. Precise mapping of genomic coordinates and generation of graphical representations were achieved based on standardized GFF annotation data, allowing the systematic interpretation of gene positional features. The function of TBtools is to extract and visualize this information [31]. After extraction, the extracted location information can be visualized on the chromosome via the graphical function of TBtools. Covariance analysis compares the order and relative positions of genes or DNA sequences in different genomes to reveal evolutionary relationships and potential functional links between them. Subsequently, duplicate genes were analyzed using MCScanX, and homology detection was performed using default parameters (E-value < 1 × 10^−5^, match size ≥ 5 genes). The covariance of GRAS transcription factor family members in Arabidopsis, apple, and sweet orange was analyzed using TBtools software [35].

### 2.5. Analysis of Gene Structure and Structural and Conserved Motifs

Gene structure analysis focuses on the composition and arrangement of genes, including the identification and localization of elements such as exons, introns, and terminators [36]. In this study, the structure of each gene was analyzed using the gene annotation file of sweet orange with TBtools software [35]. Conserved motifs are short sequence patterns that recur in biological sequences and exhibit essential biological functions. Conserved motif analysis helps reveal functional elements and evolutionary relationships in biological sequences. The coding sequences (CDSs) of the extracted *GRAS* genes were used as input files for the online software MEME (http://meme-suite.org/ (accessed on 20 July 2024)). To ensure that the CDS of each gene was submitted as one or more lines (as required by MEME), parameters were set with conserved motifs specified as 10 and others defaulted [37]. The analysis generated an output file containing information such as motif positions and E-values. Finally, the results were visualized using TBtools software [35].

### 2.6. Analysis of Cis-Acting Elements

A cis-acting element is a DNA sequence located in the regulatory region of a gene (e.g., promoter or enhancer) that modulates its expression. They do not encode any proteins themselves but provide a site of action that interacts with trans-acting factors (e.g., transcription factors) and thus participate in regulating gene expression. Based on the identification of the genes obtained, the location of the promoter is first determined (usually upstream of the CDS in the coding region of the gene), and the base sequences 2000 bp upstream of each gene can be extracted individually from the genome annotation file via TBtools software. Cis-acting element predictions were analyzed for cis-acting promoters via the PlantCARE (https://bioinformatics.psb.ugent.be/webtools/plantcare/html/ (accessed on 22 July 2024)) online software [38].

### 2.7. GO Function and KEGG Pathway Enrichment Analysis of GRAS Genes

GO function and KEGG pathway enrichment analyses of *GRAS* genes constitute an essential part of biological research to reveal the specific functions of *GRAS* genes in organisms and the metabolic pathways involved. A better understanding of the biological functions of *GRAS* gene-encoded proteins is needed. In this study, the proteins of 43 *CsGRAS* genes were functionally annotated via the online software EggNOG-MAPPER (http://eggnog-mapper.embl.de/ (accessed on 24 July 2024)) [39]. The sweet orange *GRAS* genes were subsequently analyzed for GO function and KEGG pathway enrichment via TBtools software. Plotting and visualization were performed via the online tool ChiPlot (https://www.chiplot.online/ (accessed on 25 July 2024)) [32].

### 2.8. Plant Material, Growing, and Salt Treatments

Sweet orange (*Citrus sinensis*) fruits were harvested from a commercial orchard located in Xinping County, Yunnan Province, China, and subsequently processed under controlled laboratory conditions. The laboratory environment was maintained at a constant temperature of 25 °C with a photoperiod of 16 h light (1500 lx) and 8 h darkness to simulate diurnal growth rhythms. Fruits were surface-sterilized using sodium hypochlorite (NaClO) and ethanol (75% *v*/*v*) under sterile conditions. Seeds were carefully extracted from the sterilized fruits and germinated in vitro on modified Murashige and Skoog (MS) medium supplemented with appropriate nutrients and plant growth regulators.

To investigate the effects of 2% (*w*/*v*) NaCl-induced salt stress on sweet orange seedlings, a controlled experimental design was implemented [40]. Seedlings at comparable developmental stages were allocated to either a treatment group (exposed to salt stress) or a control group (maintained under standard growth conditions). Each group comprised 12 seedlings, which were further randomized into three independent biological replicates (*n* = 4 seedlings per replicate). Technical replicates (*n* = 3) were performed for each biological replicate to ensure data reliability. Seedlings in the treatment group were continuously exposed to 2% NaCl solution for 14 days, while the control group was cultivated in standard MS medium without salt supplementation. For transcriptomic analysis, leaf tissues (1 g fresh weight) were collected from both treatment and control groups at the endpoint of the stress period. Samples were immediately flash-frozen in liquid nitrogen to halt RNA degradation and preserve transcriptional profiles. Three biological replicates per group were processed and submitted to PharmaGene Biotechnology Co., Ltd. in Suzhou, China for RNA sequencing (RNA-Seq). The resulting transcriptomic data were analyzed to elucidate global gene expression patterns and hormone metabolic pathways under salt stress conditions.

### 2.9. Transcriptome Sequencing Analysis

In transcriptome sequencing (RNA-seq) data analysis, data filtering, quality control, comparison with reference sequences, and subsequent quantitative analysis are the key steps. First, data processing and quality control involved the following: raw sequencing reads were quality assessed by FastQC v0.11.9 to evaluate base quality scores, junction contamination, and GC content distribution. Low quality bases (Phred score < 30) and residual junctions were removed using Trim Galore! v0.6.7 with stringent parameters (--quality 30 --stringency 3 --length 20). Next was alignment and quantification as follows: processed sequencing reads were aligned to the sweet orange reference genome (*Citrus sinensis* v2.0, assembly sequence GCA_002743455.1) using HISAT2 v2.2.1 (transcriptome-aware parameter: --dta --rna-strandness RF), with an average inter-sample alignment efficiency of 92.3 ± 2.1%. Gene level expression quantification was performed using featureCounts v2.0.3 (Subread package) with exon targeted counting (-t exon -g gene_id) while respecting fragment mapping constraints (-p -B -C). Finally, differential expression analysis was performed as follows: (i) Counts were normalized by the median ratio method of DESeq2 v1.38.3 to correct for library size and compositional bias, and normalized expression values (FPKM) were generated for visualization. (ii) A generalized linear model (GLM) with negative binomial distribution was used for statistical tests. Dispersion estimation was performed using default settings and apeglm shrinkage was applied to log2-fold change (LFC). (iii) Genes were considered differentially expressed when |LFC| ≥ 1 (equivalent to ≥2-fold change) and the adjusted *p*-value (Benjamini-Hochberg FDR) ≤ 0.05. Based on these criteria, 42 CsGRAS genes were identified as significantly differentially expressed under salt stress. One CsGRAS gene did not meet the significance criteria for differential expression. In that case, the statistical efficacy of this experimental design, as calculated by RNASeqPower, is 0.88 [41]. TBtools was used to construct a heatmap of sweet orange *GRAS* gene expression. Transcriptome-related data have been uploaded to NCBI (GEO accession number: GSE248845).

### 2.10. qRT-PCR Analysis

To further investigate the expression of the *CsGRAS15*, *CsGRAS27, CsGRAS35*, and *CsGRAS38* genes under salt stress, leaves from sweet orange tissue-cultured seedlings grown in normal medium were designated as the control group (CK1, CK2, and CK3), while leaves from sweet orange plants grown in a 2% NaCl concentration were used as the treatment group (T1, T2, and T3). Total RNA was extracted from the sweet orange leaves using the plant RNA extraction kit (DP432) and subsequently reverse transcribed into cDNA with the FastKing RT Kit (KR116). The reverse-transcribed cDNA was diluted 10-fold, mixed thoroughly, and utilized for the formal quantitative PCR experiment. Actin was used as the internal reference gene, and qRT-PCR primers were designed using Primer 6.0 (Table S1). Standard RT-qPCR was conducted with the QIAGEN kit, with each gene analyzed in at least three replicates. Data analysis was performed using SPSS v21 software.

## 3. Results

### 3.1. Identification and Physicochemical Characterization of the Sweet Orange GRAS Transcription Factor Family

**Figure 1:**
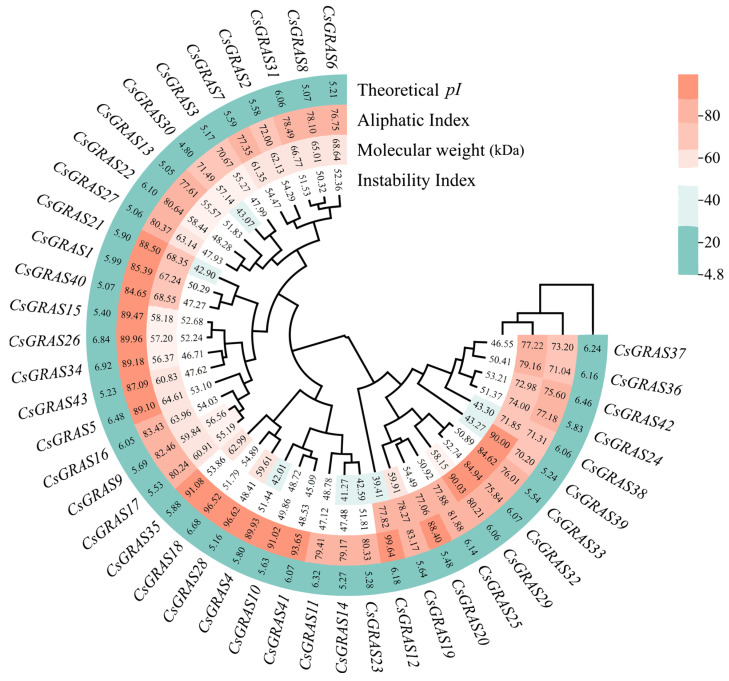
Physicochemical property analysis of the sweet orange *GRAS* gene heatmap. The numbers in the heatmap indicate numerical values, with larger values being darker; the outermost circle indicates the gene’s name, followed by the theoretical pI, aliphatic index, molecular weight (kDa), and instability index.

Using known GRAS transcription factor sequences or conserved motifs as query sequences, BLAST and hmm searches were performed in the Sweet Orange Genome Database to identify potential *GRAS* gene family members, after which the Sweet Orange GRAS transcription factor families were predicted via CDD and Pfam analysis. The final results can be obtained by removing incomplete or duplicate gene family members. Forty-three *GRAS* genes were identified in the whole genome of sweet orange (Figure 1). We renamed the genes according to their order on the chromosome, from top to bottom, as *CsGRAS1*-*CsGRAS43* (Table S2). Further analysis of their sequences revealed that the individual GRAS transcription factor proteins do not differ much in length, with amino acid numbers ranging from 424 (*CsGRAS11*)-749 (*CsGRAS33*) amino acids; molecular weights ranging from 47.12 (*CsGRAS11*)-90.03 (*CsGRAS29*) kDa; *pI* ranging from 5.05 (*CsGRAS13*)–6.92 (*CsGRAS34*); and instability coefficients ranging from 39.41 (*CsGRAS12*)–62.99 (*CsGRAS35*). The amino acid composition analysis showed that spherical proteins had an average aliphatic index of 82, predicting higher thermal stability. Subcellular localization prediction using the Plant-mPLoc tool indicated that all proteins were located in the cell nucleus (Table S3).

### 3.2. Sequence Similarity and Phylogenetic Analysis of the Sweet Orange GRAS Gene Family

**Figure 2:**
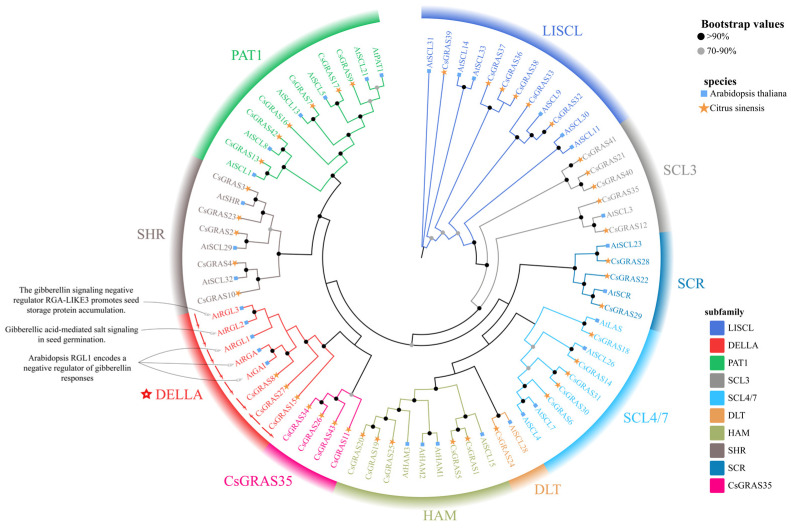
Phylogenetic tree of sweet orange and *A. thaliana* GRAS transcription factor family genes. This phylogenetic tree was constructed via the maximum likelihood method with a total bootstrap value of 1000 replicates; black indicates that the self-expansion value is higher than 90% in 1000 replicates; grey indicates that the self-expansion value is between 70% and 90% in 1000 replicates; and the higher the bootstrap value is, the more frequently the branch appears stably in, the higher the bootstrap value is, the higher the frequency of stable occurrence of the branch in multiple randomly rearranged trees, and therefore, the higher the confidence. The green triangles represent the species *A. thaliana*, and the blue stars represent sweet orange. Different colors indicate different subfamily groups. The five red stars represent subfamilies with specific resistance, and the red arrows indicate positive and negative expression of genes under salt stress. The main biological functions of some GRAS proteins and some representative references are shown in the phylogenetic tree.

To analyze the homology among *CsGRAS* gene family members, this study used ClustalW and showed that protein sequences within the same subfamily exhibit a high similarity (Supplementary Figure S1). For example, *CsGRAS8*, *CsGRAS15*, and *CsGRAS27* in the DELLA subfamily share a similarity of up to 87.65%. To better understand the evolutionary relationships between sweet orange GRAS transcription factor family genes and those of other species, 43 sweet orange GRAS protein sequences and 32 GRAS protein sequences of *A. thaliana* were compared, and a phylogenetic tree was constructed. The phylogenetic tree was built using MEGA and IQ-TREE software via the maximum likelihood (ML) method for the 43 sweet orange *GRAS* genes and 32 *A. thaliana GRAS* genes, allowing the identification of *GRAS* gene differences across species. The results revealed a consistent tree topology (Figure 2), indicating that the 43 sweet orange and 32 *A. thaliana GRAS* genes were classified into 10 subfamilies, including LISCL, SCL3, SCR, SCL4/7, DLT, HAM, CsGRAS35, DELLA, SHR, and PAT1. Under salt stress, KEGG enrichment analysis identified only one GA phytohormone signaling pathway, where DELLA proteins play a vital regulatory role. The results showed that all five Arabidopsis genes in the DELLA subfamily have specific salt-resistance functions, suggesting that sweet orange *GRAS* genes in the DELLA subfamily may exhibit similar expression patterns under salt stress. RGL2 [42] and RGL3 [43] are DELLA proteins that negatively regulate GA signaling, while RGA is a negative regulator of the GA response; RGL1 has a more pronounced effect on seed germination than GAI, and both RGA and RGL2 negatively regulate GA signaling [44]. Among the three differentially expressed genes, *CsGRAS8*, *CsGRAS15*, and *CsGRAS27* (which belong to the same subfamily as these Arabidopsis genes) are significantly differentially expressed, with *CsGRAS8*/*CsGRAS15* downregulated and *CsGRAS27* upregulated, respectively.

### 3.3. Chromosomal Localization, Gene, and Covariance Analysis of the Sweet Orange GRAS Transcription Factor Family

**Figure 3:**
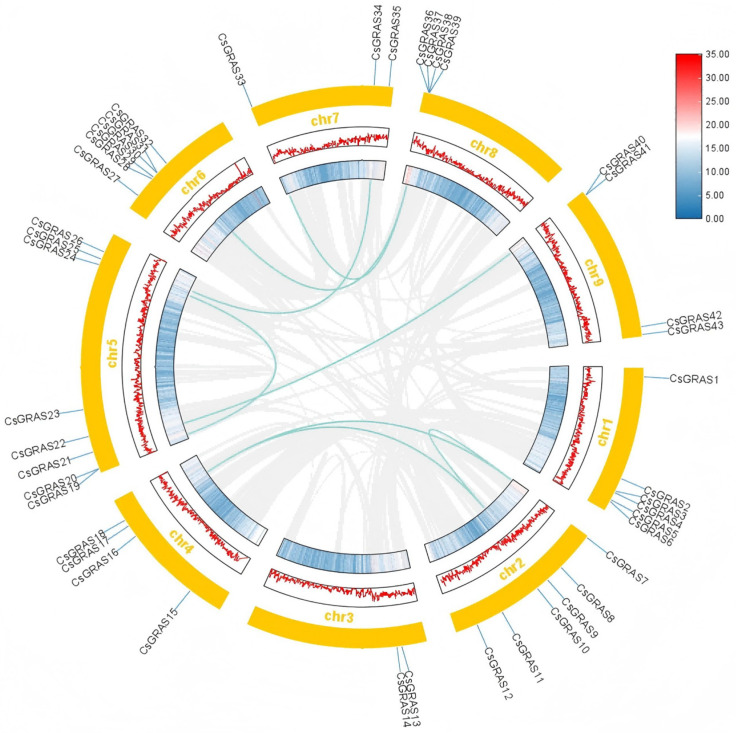
Covariance analysis of sweet orange *GRAS* genes. The grey line represents all duplicated genes, and the dark green line indicates segmentally duplicated *GRAS* gene pairs. Heat and line plots represent gene density, with line density increasing from blue to white to red, and yellow rectangles are chromosomes on which chromosome names are displayed.

The localizations of 43 CsGRAS genes on nine chromosomes of the genome of sweet orange were analyzed via TBtools software (Figure 3). The results revealed that the CsGRAS genes were present on all nine chromosomes, with the highest number of CsGRAS genes distributed on chromosome chr5, with eight. Chr4, 8, and 9 all had four CsGRAS genes distributed on chromosomes chr4, 8, and 9. Three to six CsGRAS genes were distributed on the remaining chromosomes, with uneven distribution and no direct correlation between chromosome length and gene number.

Gene duplication is the increase in the number of copies of a gene sequence in the genome [40]. This duplication can involve an entire gene, a partial gene fragment, or even an entire chromosome or genome. Fragment duplication is the primary driver of gene duplication [45]. To elucidate the evolutionary duplication patterns within the *CsGRAS* gene family of sweet orange, we conducted a genome-wide duplication analysis encompassing all 43 identified *CsGRAS* genes. This analysis aimed to reveal the copy number variations of *CsGRAS* genes in the sweet orange genome. There were eight segmental duplication gene pairs, and the segmental duplication gene pairs were distributed on six chromosomes, with the highest number of genes distributed on chromosome 5, which was four. On the remaining chromosomes, 2, 1, 1, 2, and 1 *CsGRAS* genes were distributed on chromosomes chr2, 4, 6, 7, and 8, respectively.

**Figure 4:**
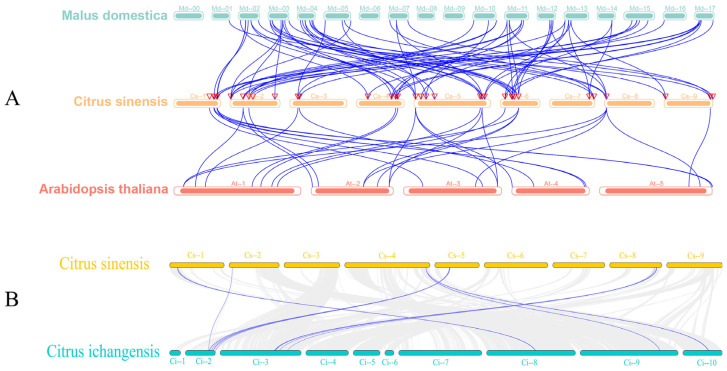
Covariance map of *GRAS* genes in sweet orange (**A**): Collinearity plot between different species of sweet orange, Arabidopsis thaliana, and apple; blue lines indicate collinear *GRAS* gene pairs. At-, Cs-, and Md- denote the chromosomes of *A. thaliana*, sweet orange, and apple, respectively. (**B**): Collinearity plot between sweet orange and its close relative Yichang orange, grey lines denote the duplicated blocks, whereas blue lines denote collinear *GRAS* gene pairs. Cs- and Ci- denote the chromosomes of the sweet orange and Yichang orange chromosomes, respectively.

An interspecies synteny analysis of *GRAS* genes among apple (*Malus domestica*), sweet orange (*Citrus sinensis*), and Arabidopsis (*Arabidopsis thaliana*) was performed (Figure 4A). The interspecies syntenic analysis revealed a greater collinearity between sweet orange and its distantly related species (apple) than between sweet orange and the model species (*A. thaliana*), indicating a more remarkable similarity in genome architecture between sweet orange and apple, which may suggest a closer evolutionary relationship between these two species. Furthermore, synteny analysis between sweet orange and its closely related species, Yichang orange (*Citrus ichangensis*), was conducted (Figure 4B). The study demonstrated that homologous gene pairs between sweet orange and Yichang orange significantly outnumbered those observed in the other two species, confirming their closer evolutionary relationships than those of more distantly related taxa.

### 3.4. Analysis of the Gene Structure and Structural and Conserved Motifs of the Sweet Orange GRAS Transcription Factor Family

**Figure 5:**
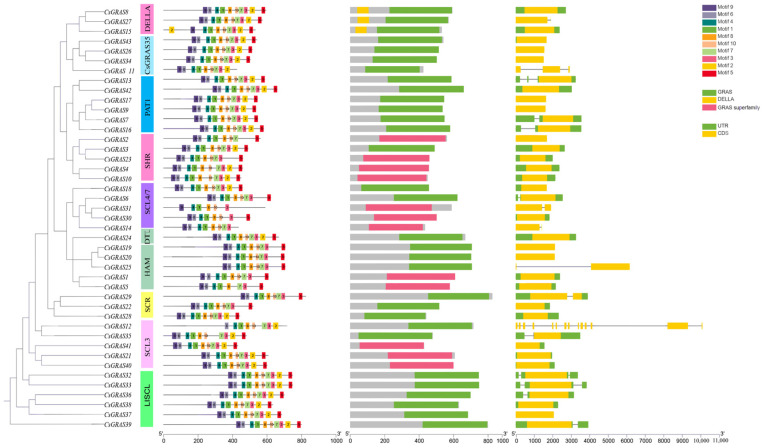
Phylogenetic relationships, gene structure, and structural and conserved motif distributions of CsGRAS transcription factor family genes. Phylogenetic tree analysis of 43 GRAS transcription factor family genes constructed by MERGA; conserved motif distribution of *CsGRAS* genes; colored boxes indicate the motifs; black lines indicate the relative length of the proteins; conserved structural domains of the *CsGRAS* genes; and the exon–intron structure diagram of the *CsGRAS* gene, where green indicates UTR, yellow indicates CDS, and the middle line segment indicates introns.

A conserved motif is a specific pattern or sequence fragment that occurs frequently and remains relatively stable in a biological sequence (e.g., DNA, RNA, or protein sequence) [46]. In protein sequences, a conserved motif can be a specific arrangement of one or several amino acid residues that may form a particular secondary structure (e.g., α-helix, β-folding) or participate in specific interactions (e.g., hydrogen bonding, ionic bonding, hydrophobic interactions, etc.) in a three-dimensional structure. These conserved amino acid sequences and structural features enable proteins to perform their specific biological functions [47]. To understand the function and structure of proteins encoded by GRAS genes, this study used the MEME online tool to analyze and obtain the motif composition of CsGRAS proteins (Figure 5). Motif 2 appeared twice in the protein sequence of *CsGRAS15*, suggesting that the *CsGRAS15* gene in the DELLA subfamily may be involved in particular functions. In the PAT1 subfamily, despite generally exhibiting a high degree of motif conservation across all core motifs, a deeper look reveals that even within this closely related group of members, the motif distribution exhibits subtle variability. Similar to *CsGRAS15*, motif 2 appears twice in its protein sequence, and this atypical motif duplication may reveal the evolutionary strategies of transcription factor family genes that occur under specific environments or developmental stages to adapt to different regulatory needs. These findings may imply that these genes play more complex or particular roles in the transcriptional regulatory network, enhancing or fine-tuning their interactions with other regulatory elements by increasing the copy number of motif 2 to achieve precise regulation of target gene expression.

The analysis of gene structure allows the determination of sequence variations, intron–exon organization patterns, and the retention and evolution of specific structural domains that have accumulated during evolution among different CsGRAS transcription factors. The sequences of 43 CsGRAS transcription factors were compared, and their gene structures were analyzed using genome annotation files. The number of introns and exons in sweet orange GRAS genes showed little variation, with intron counts ranging from 0 to 13 and exon counts from 1 to 14. Additionally, this study deeply explored the properties of conserved structural domains, revealing that all 43 CsGRAS proteins contain either GRAS or GRAS superfamily domains. A particularly notable finding is the unique DELLA domains present in members of the DELLA subfamily clade, which play an indispensable role in signal transduction [48].

### 3.5. Analysis of Critical Cis-Acting Elements in the Sweet Orange GRAS Promoter

**Figure 6:**
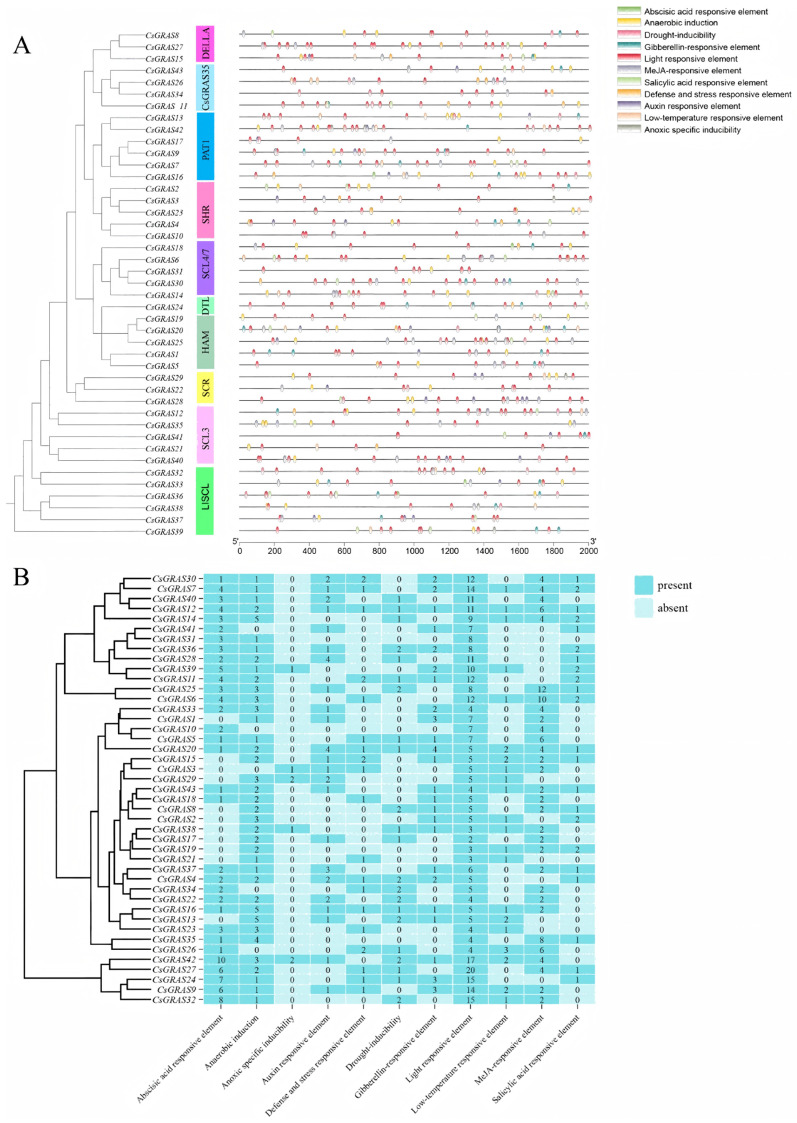
Cis-acting elements of sweet orange *GRAS* genes. (**A**) Position of each cis-acting element. (**B**) The number of cis-acting elements of each *GRAS* gene, and the numbers in the heatmap boxes indicate the number of different elements in these *GsGRAS* genes.

The upstream sequences (2000 bp) were extracted based on the chromosomal location of the *GRAS* genes. The PlantCARE plant cis-acting element analysis tool was used to examine the cis-acting elements in the target sequences. The results revealed that the promoter region of each *CsGRAS* gene has multiple associated cis-acting elements (Figure 6A). These specific DNA elements, which are based on their functions, are systematically classified into four core categories: First, plant developmental and growth regulatory elements, which are responsible for regulating the developmental process and growth rate of plants at various stages of their life cycle; second, plant hormone response elements, which can recognize and respond to the signals of different hormones in the plant body and thus regulate the expression of relevant genes to maintain the plant’s internal characteristics of the plant; and third, light-responsive elements, which are highly sensitive to light signals such as light intensity, direction and duration and regulate the expression of photosynthesis-related genes and other light-dependent genes to ensure that plants can efficiently utilize light energy to carry out their life activities. Finally, abiotic stress response elements, which play a key role when plants face unfavorable environmental conditions such as drought, high salt, low temperature, and high temperature, help plants resist and adapt to these abiotic stresses by regulating the expression of resistance-related genes. Using the PlantCARE online software [38], the sequences of the upstream 2000 bp regions of 43 *GRAS* gene promoters in sweet orange were analyzed for cis-acting elements. The results (Figure 6B) showed that a total of 820 cis-acting elements were identified, including 331 light-responsive elements, 319 plant hormone-responsive elements, 163 environmental stress-responsive elements, and 7 plant-specific regulatory elements. Trans-acting elements were primarily categorized into four types. The proportions of trans-acting response elements were 40.37%, 38.90%, 19.88%, and 0.85%, respectively. Among the 43 gene family members, the maximum number of trans-acting response elements was 20, and the minimum was 0. In summary, excluding light-responsive elements, as many as 54.8% of the promoter regulatory elements play a key role in plants.

In addition, the DELLA protein is a negative regulator of the GA signaling pathway. Light can effectively inhibit the accumulation of the DELLA protein in germinating seeds. In contrast, salt stress promotes the accumulation of the DELLA protein and the increase in the DELLA protein, thus inhibiting the growth and development of rhizomes to regulate the resistance of sweet orange plants under salt stress. Therefore, it is crucial to have light-responsive action elements in sweet orange *GRAS* genes; as we can see from the number of cis-action elements of each *GRAS* gene, the three *CsGRAS* genes in the DELLA subfamily and their light-responsive action elements are 5, 5, and 20, respectively, which are in the middle to upper level compared with the number of other *CsGRAS* genes. Compared with the number of other *CsGRAS* genes, the number of light-responsive elements in the *CsGRAS27* gene is between the middle and upper levels. Notably, *CsGRAS27* harbors the highest number of light-responsive elements overall, indicating heightened light sensitivity and the ability to effectively regulate DELLA protein levels in response to light intensity changes.

### 3.6. GO Functional Annotation and KEGG Enrichment Analysis of the Sweet Orange GRAS Transcription Factor Family

**Figure 7:**
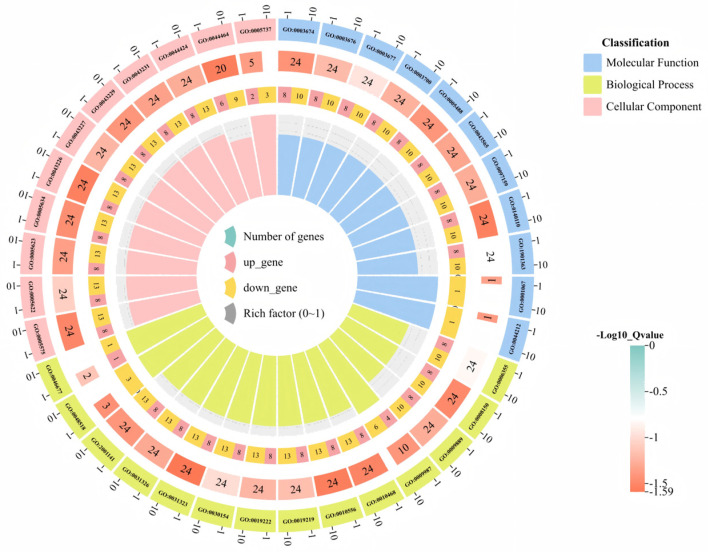
GO functional analysis of *GRAS* genes under salt stress in sweet orange. From the inside out, the 1st circle bar graph represents the ratio of the number of *GRAS* DEGs enriched with the same GO term to the total number of *GRAS* genes; the 2nd circle represents the ratio of the number of *GRAS* genes enriched with differences in the up- and downregulation of the GO terms, with the pink color indicating upregulation and the darker yellow color indicating downregulation. The 3rd circle heatmap represents the total number of genes enriched on the corresponding GO term. The 4th circle represents the GO number, and different classifications are indicated by different colors.

**Figure 8:**
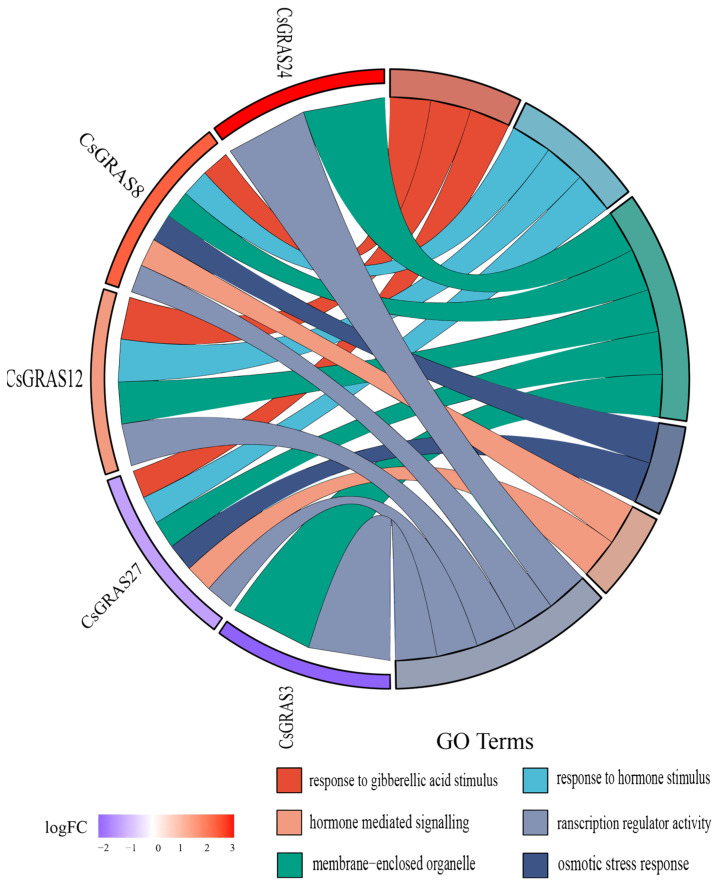
Expression patterns and functional annotations of genes with specific anti-salt stress functions under salt stress. The GO chord diagram is divided into two parts, with the middle as the boundary. On the left side are the genes arranged according to logFC, and on the right side are the GO terms, with different colors indicating different functions.

Gene Ontology (GO) and Kyoto Encyclopedia of Genes and Genomes (KEGG) pathway enrichment analyses are indispensable tools in bioinformatics research. In this study, functional annotation of the 43 CsGRAS transcription factors was performed using eggNOG-mapper. The results (Figure 7, Table S4) revealed that 23 *CsGRAS* genes were annotated across three primary GO categories: Biological Process (BP), Cellular Component (CC), and Molecular Function (MF). Within Molecular Function, *CsGRAS* genes were significantly enriched in DNA-binding transcription factor activity, sequence-specific DNA binding, and organic cyclic compound binding. This enrichment profile suggests these genes likely possess specific functional roles or high activity, potentially participating in coordinated biochemical processes or enzymatic reactions. For Cellular Component, *CsGRAS* genes showed enrichment in terms associated with intracellular anatomical structures, membrane-bounded organelles, and intracellular components. This localization pattern implies potential involvement in critical biological processes, such as cell division, substance transport, and signal transduction. Regarding Biological Process, significant enrichment was observed for processes including biosynthesis, the regulation of gene expression, and macromolecule biosynthetic regulation. These processes are fundamental to organismal survival, reproduction, development, and environmental adaptation. Furthermore, to elucidate the roles of salt-stress-associated *CsGRAS* genes, a GO chord diagram (Figure 8) was constructed based on the enrichment results. This analysis identified three *CsGRAS* genes exhibiting significant differential expression linked to salt stress. Notably, *CsGRAS27* (a member of the DELLA subfamily) was among these genes. Given that genes within the same subfamily often share functional pathways, this finding suggests *CsGRAS15* (also a DELLA subfamily member) may participate in analogous metabolic or signal transduction pathways in response to salt stress.

**Figure 9:**
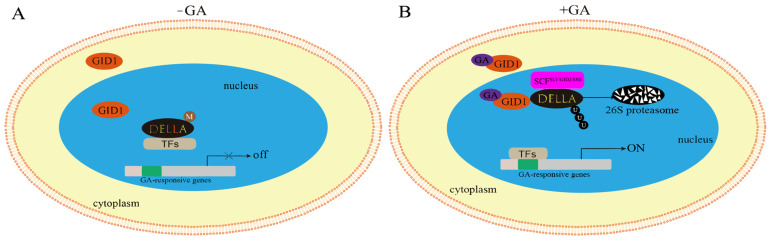
Model of DELLA protein-mediated GA signaling. (**A**) DELLA protein pathway response in the absence of gibberellin action. (**B**) Activation of the DELLA protein pathway under the influence of gibberellic acid.

KEGG pathway enrichment analysis maps differentially expressed genes (DEGs) to established metabolic and signaling pathways (Table S5). This helps us to understand how genes are involved in specific metabolic and signaling processes in organisms [49]. The KEGG enrichment analysis revealed that only 5 of the 43 *GRAS* genes were enriched for unique phytohormone signaling pathways, of which *CsGRAS8*, *CsGRAS15*, and *CsGRAS27* belong to the DELLA subfamily, which are all genes in this subfamily. The remaining two genes belong to the SCL3 subfamily. In addition, a model of GA signaling mediated by DELLA proteins under normal growth conditions (in the absence of stress) was constructed via KEGG enrichment analysis (Figure 9). As shown in Figure 9B, DELLA proteins act as negative regulators in the GA signaling pathway to deter plant growth and development. After active GA binds to GID1 in the cytoplasm, it then promotes the binding of GID1 to the N-terminus of the DELLA protein in the nucleus, leading to a conformational change in the DELLA protein and promoting the interaction of the C-terminus of the DELLA protein with SCF^SLY/GID2/SNE^ (the SCF complex is an E3 ubiquitin ligase in the 26S proteasome degradation system). This results in the formation of the GID1-GA-DELLA protein complex, which enhances the interaction between DELLA and SCFSLY/GID2/SNE, leading to the ubiquitination of the DELLA protein and its degradation via the 26S proteasome complex, which in turn relieves the deterrent effect of the DELLA protein.

### 3.7. Analysis of Protein Network Interactions of the Sweet Orange GRAS Transcription Factor Family

**Figure 10:**
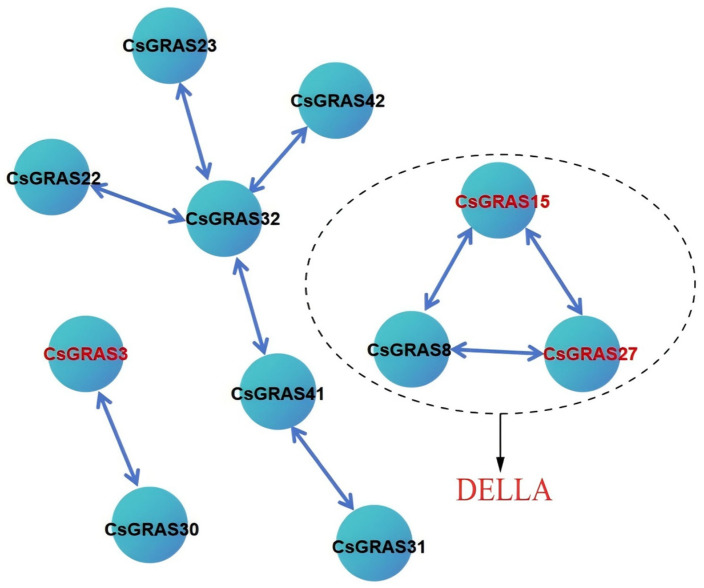
PPI interaction diagram; each circle represents a protein.

Network interactions of 43 CsGRAS proteins were analyzed, and the results (Figure 10) showed that 13 genes formed four different interaction groups. Among the 43 CsGRAS proteins, the remaining 30 genes did not exhibit interactions, indicating that interacting proteins play critical roles. Notably, *CsGRAS8*, *CsGRAS15*, and *CsGRAS27*—members of the DELLA subfamily—form interactions and are known to play central roles in the GA signaling pathway [50], highlighting the inseparable link between the functions of these three genes and the DELLA subfamily.

### 3.8. Expression Pattern Analysis of CsGRAS Transcription Factor Family Genes Under Salt Stress

**Figure 11:**
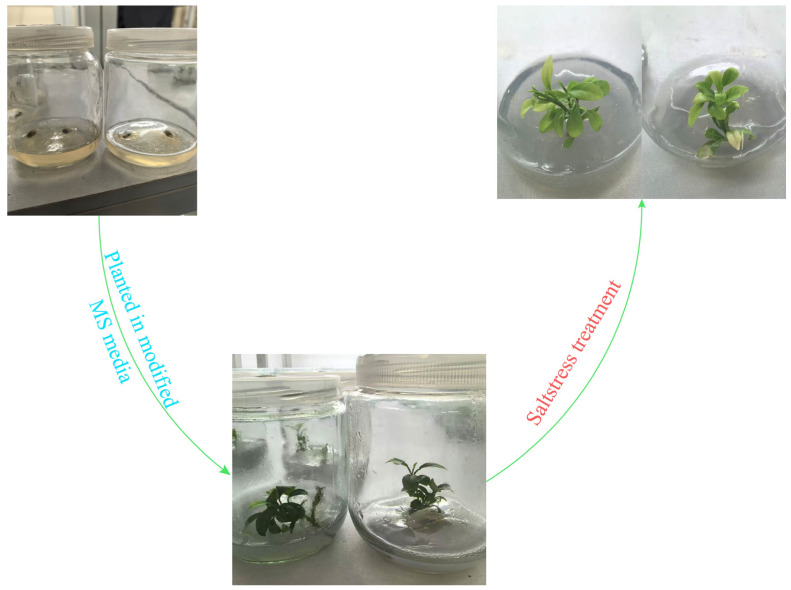
Salt stress experimental treatment chart.

**Figure 12:**
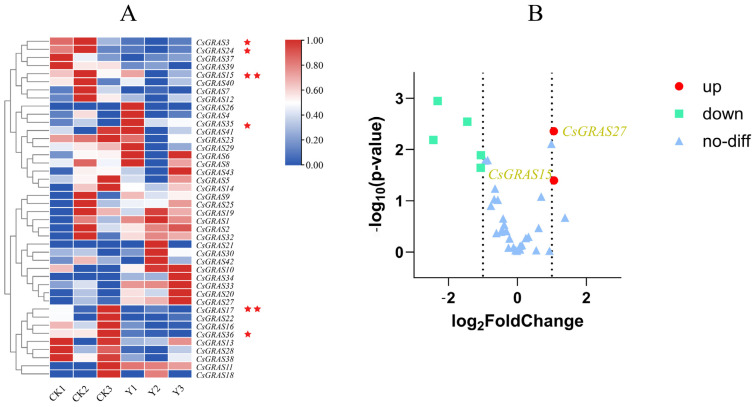
Presents a heatmap illustrating the expression profiles of 42 *CsGRAS* genes in response to salt stress. Panel (**A**) displays the heatmap of differentially expressed *CsGRAS* genes under salt stress; the intensity of red indicates higher expression levels, while the intensity of blue indicates lower expression levels. CK represents the control group, and Y represents the treatment group. Red stars indicate significantly different genes. (**B**): Volcano plot of the transcriptome; the *x*-axis represents the differential fold change in gene expression (log_2_(fold change, log_2_FC)). The *y*-axis represents the statistical significance of differential expression (−log_10_(*p*-value) or −log_10_(adjusted *p*-value). The higher the value is, the greater the importance (the smaller the *p*-value)). Red circles indicate significantly differentially upregulated genes; green squares indicate significantly downregulated genes; and light-blue triangles indicate differentially expressed genes.

When plants are in an adversarial environment, their cells display adaptations that respond to dramatic fluctuations in external conditions by continuously adjusting their gene expression patterns. At the core of this complex adaptation process lies a multilevel regulatory network constructed of transcription factor families [51]. Following a two-week salt stress treatment, plants exhibited characteristic symptoms of salt-induced injury. These included progressive leaf yellowing, marginal leaf necrosis, and the curling of young leaves. Notably, the observed leaf yellowing may reflect photoprotective chlorophyll degradation, an initial stress response, or potentially indicate the onset of programmed cell death (PCD) in severely damaged tissues. These phenotypic alterations are consistent with established markers of ionic toxicity and oxidative damage under salt stress conditions (Figure 11). To understand the expression patterns of *CsGRAS* genes in sweet orange under salt stress, we conducted a transcriptome analysis of differential gene expression, identifying 42 expressed *CsGRAS* genes. Among these, 19 genes were up-regulated, while 23 genes were down-regulated. Compared with those in the control group, seven genes were significantly differentially expressed (Figure 12A, Table S6), namely, *CsGRAS3*, *CsGRAS15*, *CsGRAS16*, *CsGRAS24*, *CsGRAS27*, *CsGRAS35*, and *CsGRAS38*. Among the seven DEGs, only the genes encoding *CsGRAS27* and *CsGRAS35* were upregulated.

**Figure 13:**
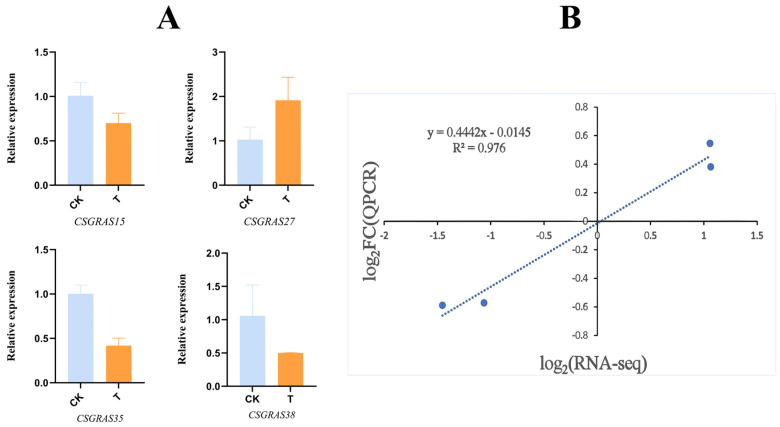
qRT-PCR validation of significantly differentially expressed CsGRAS genes. All data are averages of three biological replicates. (**A**) qRT-PCR validation of four CsGRAS genes that exhibit significant differential expression in sweet orange. (**B**) Correlation analysis between transcriptomic data and qRT-PCR data, with error bars representing the standard deviation of these replicates.

To further verify the accuracy of the transcriptome data, four *CsGRAS* genes exhibiting significant differences in the transcriptome data were selected for qRT-PCR validation. As shown in the results (Figure 13A, Table S7), the expression levels of *CsGRAS15*, *CsGRAS27*, *CsGRAS35*, and *CsGRAS38* were either up-regulated or down-regulated following salt stress when compared to the control group (CK). A correlation was found between the transcriptomics data and real-time quantitative PCR data. The dashed line represents the linear regression fit (R^2^ = 0.976), with a residual standard deviation of 0.35 (Figure 13B). These findings indicate that the RNA-seq data are reliable, as qRT-PCR further confirms the accuracy of the transcriptome data, demonstrating a high correlation with the transcriptome results. Collectively, these results validate the accuracy of the transcriptomic data and reinforce the reliability of the differentially expressed genes identified within the transcriptome. Additionally, they suggest that these *CsGRAS* genes play a crucial role in the response of sweet orange to salt stress.

### 3.9. Multiple Sequence Alignment and Protein Tertiary Structure Prediction of DELLA Subfamily Genes

**Figure 14:**
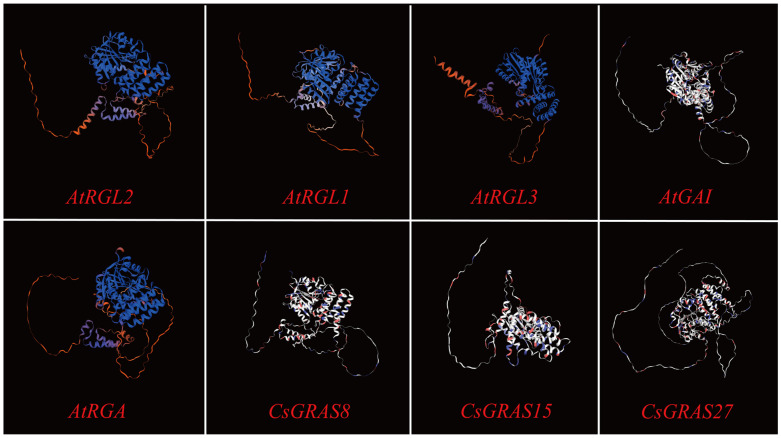
Tertiary structure of sweet orange and Arabidopsis GRAS proteins in the DELLA subfamily.

In this study, only the genes in the DELLA subfamily were analyzed via multiple sequence alignment (important parts were intercepted). Multiple sequence alignment (Supplementary Figure S2) revealed that most CsGRAS proteins have n-terminal and highly conserved C-terminal ends, and the conserved domains located at the C-terminal end include VHIID, LHRI, and SAW. In the entire DELLA subfamily, the 12 proteins included all the contained VHIID and LHRI structural domains. The VHIID domains, together with other regions (e.g., LHRI, PFYRE, and SAW), are involved in protein interactions that regulate plant growth and development and gibberellin signaling [52]. Proteins with similar sequences have similar three-dimensional structures, and the same structural domains in different proteins have identical functions. The protein tertiary structures of sweet orange and Arabidopsis *GRAS* members in the DELLA subfamily were predicted via SWISS-MODEL (Figure 14), and the GMQE values ranged from 0.49 (*AtRGL1*) to 0.85 (*CsGRAS15*); the tertiary structure analyses revealed that the structures were very similar among the subfamily members. The high structural similarity between the sweet orange *GRAS* members in DELLA and the Arabidopsis *GRAS* members further suggests that both may have the same function.

### 3.10. Analysis of Gibberellin Signaling Under Salt Stress

**Figure 15:**
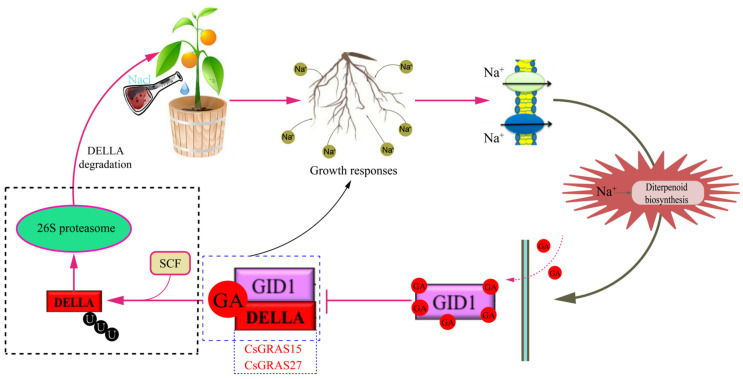
Diagram of the KEGG phytohormone signaling pathway under salt stress in which the *CsGRAS15* and *CsGRAS27* genes act. Under salt stress, sodium ions penetrate the cell membrane to reach the nucleus, promoting diterpenoid biosynthesis and thereby directing the binding of GA molecules to the receptor GID1, which subsequently enhances the affinity of GID1 for the N-terminus of the DELLA protein, triggering a structural change in the DELLA protein (i.e., within the blue dashed line), which inhibits the overgrowth of the root to acclimatize to and resist salt stress. Under normal conditions (i.e., black dashed line), DELLA acts as a negative regulator in the GA signaling pathway, inhibiting plant growth and development. Suppose that the interaction between the DELLA and the SCF is enhanced. In this case, the DELLA protein is ubiquitylated and degraded by the 26S protease complex, which in turn removes the inhibitory effect of the DELLA protein, and the plant can ultimately develop normally.

The analysis revealed that the *CsGRAS15* gene has no GO annotation function but has a KEGG enrichment function. The KEGG enrichment analysis under salt stress conditions revealed (Figure 15) that genes in the DELLA subfamily of the sweet orange GRAS transcription factor family encode DELLA proteins, which are negative regulators of the GA response and play vital roles in the plant response to gibberellin (GA) signaling. Under adverse conditions such as salt stress, GA molecules first bind to the GID1 receptor, and this binding event subsequently enhances the affinity of GID1 for the N-terminus of the DELLA protein, triggering a structural change in the DELLA protein. This structural change significantly inhibited the interaction between the DELLA protein and GID2, in turn blocking the pathway that might otherwise lead to the ubiquitination of the DELLA protein. Ubiquitination is normally a signaling process that marks proteins for degradation, but in this scenario, the stability of the DELLA protein is increased as its ubiquitination is blocked. Moreover, as the DELLA protein dissociates from certain transcription factors, growth and developmental regulatory mechanisms that DELLA proteins would otherwise inhibit are released. Specifically, this change inhibited root overgrowth, allowing the plant to adjust its growth strategy to adapt to and resist adversity under unfavorable environmental conditions such as salt stress, thus ensuring survival and reproduction.

In contrast to the model of DELLA protein-mediated GA signaling under normal conditions, the degradation of DELLA proteins mediated through SCFSLY/GID2/SNE is not required in the GA signaling pathway under salt stress. When a functional DELLA structural domain is present, the inhibitory effect of DELLA proteins can be removed by GA and GID1 proteins alone.

## 4. Discussion

Soil salinization, driven by climate change, inappropriate irrigation, and coastal agricultural expansion, is a serious constraint on sweet orange production and exacerbates this threat. Unlike some wild or hybrid citrus relatives, sweet oranges—domesticated for a long time to prioritize fruit quality traits—are more sensitive to salt stress, often at the expense of stress resistance [40]. Excessive Na^+^ and Cl^−^ accumulation disrupts osmotic balance, impairs photosynthetic efficiency, induces oxidative damage, and ultimately reduces fruit yield and quality [53]. Furthermore, improving salt tolerance in sweet oranges is crucial for sustainable agriculture. Most commercial cultivars are grafted onto rootstocks with limited salt adaptation. Investigating the molecular mechanisms of salt responses in the scion-rootstock system could guide breeding programs or biotechnological interventions to expand cultivation into marginal lands. This aligns with the urgent need to mitigate food security risks under escalating environmental pressures.

The GRAS transcription factor family, which is large and critical in the plant kingdom, can be involved in the regulatory gene expression network in response to many complex and variable adversity challenges. Several studies have shown that members of this family exhibit irreplaceable functions in mitigating drought, resisting salt stress, and adapting to abiotic stresses such as low temperatures. These are important components in plant survival strategies. In addition, GRAS transcription factors regulate plant growth and development [4,48], leaving the imprint of their roles from cell division to tissue differentiation to the shaping of overall morphology. These findings not only deepen our understanding of plant stress tolerance mechanisms but also provide valuable genetic resources and a theoretical basis for future improvements in crop stress tolerance and yield via genetic engineering. The GRAS transcription factor family is named after its unique *GRAS* structural domain (an acronym for the three proteins GAI, RGA, and SCR), a conserved amino acid sequence usually located at the N-terminal end of proteins. Members of the *GRAS* family are widely found in the plant kingdom and have many biological functions. In the present study, we identified 43 *CsGRAS* genes, which is relatively high compared with the 34 Arabidopsis *GRAS* genes but lower than the number of poplar (106) [23], soybean (117) [26], and wheat (153) [54] genes, suggesting that the GRAS transcription factor family is related to the genome size of different species. By phylogenetic analysis, *CsGRAS35* emerged as a subfamily, suggesting that it may have unique functions. Other *CsGRAS* genes were observed to form tight clusters with *GRAS* genes from the model plant *A. thaliana*. This phenomenon suggests a high degree of evolutionary conservation of these genes [23]. More significantly, the corresponding sweet orange CsGRAS proteins were successfully identified in each *GRAS* subgroup of Arabidopsis. These findings further support the fundamental biological roles that these genes may have played in the long-term evolutionary history of sweet oranges. The GRAS transcription factors of sweet orange can be classified into 10 subfamilies, of which LISCL and PAT1 are the largest subfamilies; however, the SCL3, SCR, SCL4/7, HAM, DELLA, and SHR subfamilies also contain several genes with some differences. In addition, the rationality of the classification was further confirmed when the results of the conserved motif, gene structure, and structural domain analyses were considered. Specifically, the conserved motif analysis revealed that similar genes were more inclined to cluster within the same subfamily. This phenomenon not only justified the classification but also implied the potential functional similarity of these genes. Moreover, multiple motifs were found in specific proteins, which were combined in unique and consistent patterns, suggesting that these proteins may carry specific biological functions that are essential for maintaining the growth and development of sweet oranges and their ability to adapt to the environment [55]. It is important to note that, despite our efforts to enhance resolution through subfamily analysis and experimental validation, the enrichment methods based on annotation databases for the functional prediction of large protein families may still be affected by functional redundancy among homologs. Future studies that integrate single-cell expression profiles or deep learning predictions, such as AlphaFold structural-functional inference, may further refine these conclusions. In the analysis of structural domains, the DELLA structural domains were found to appear exclusively in the DELLA subfamily, whereas the GRAS superfamily was identified only in SHR, SCL4/7, HAM, and SCL3. This finding not only reveals the complexity of the internal structure of the *GRAS* gene family but also suggests that this heterogeneity in the distribution of the structural domains enables the diversity of the *GRAS* gene family to develop and profoundly influence its functional differentiation and specialization. Furthermore, our gene structure analysis revealed that most of the subfamily genes are intronless, except for the PAT1, SCL3, and LISCL subfamily CsGRAS genes, which are similar to *GRAS* genes in sweet potato [56], *A. thaliana* [21], and potato [57]. This finding illustrates that intronless genes tend to respond quickly to changes in environmental conditions [58]. However, the *CsGRAS12* gene in the SCL3 subfamily contains 13 introns, which is significantly different from other *CsGRAS* genes, and this difference in the number of introns is likely a result of chromosomal structural variation, which may further contribute to the functional diversity within the gene family. Specifically, the variation in the number of introns may affect the transcriptional regulation of the gene, mRNA stability, or translation efficiency, thus conferring unique biological functions to the *CsGRAS12* gene and enriching the functional spectrum of the entire *CsGRAS* gene family. In summary, the clustering pattern of the phylogenetic tree shows a high degree of consistency with the structural features of conserved motifs, gene structures, and structural domains, which reveals that similar genes in the course of evolution often tend to possess similar functions. This pattern not only deepens our understanding of the mechanism of gene evolution but also helps to screen *GRAS* genes with similar functions. For example, heterologous overexpression of HcSCL13 in salt spicewood enhanced plant growth and salt tolerance in transgenic Arabidopsis [31]. The *A. thaliana* SCL15 gene improved the drought tolerance of plants [59]. It is speculated that PAT1 and the HAM subfamily of *CsGRAS* genes have similar functions.

Cis-acting elements play crucial roles in the transcriptional regulation of neighboring genes, and they can directly influence and regulate the transcriptional activities of these genes. In this study, the cis-acting elements in the initiation region of the CsGRAS transcription factor family were mainly light-responsive cis-acting elements, hormone-associated cis-acting elements, and environmental stress-responsive elements. Light-responsive cis-acting elements and hormone-associated cis-acting elements were more numerous, accounting for 79.27% of the total, and hormone cis-acting elements, such as abscisic acid (ABA), gibberellin (GA), methyl jasmonate (MeJA), and salicylic acid (SA) were more numerous. GA-responsive elements had the highest distribution in the LISCL subfamily, with 8 ABA-responsive elements more frequently distributed in the PAT1 and LISCL subfamilies, with 21 and 20 ABA-responsive elements, respectively. Members of the GRAS transcription factor family contain a DELLA protein structural domain at their N-terminus, which plays a crucial role in GA signaling [60]. By participating in the regulation of GA signaling pathways, DELLA proteins can influence and fine-tune multiple aspects of plant growth and development. GA levels or signaling can also affect plant salt tolerance during salt stress [61], and the absence of DELLA repressors reduces salt tolerance in Arabidopsis [62]. ABA is commonly associated with the promotion of plant growth processes, such as stem elongation, flower development, and seed germination processes. ABA acts more as a stress hormone and is crucial when plants face unfavorable environments like drought and high salt. Under normal conditions, the presence of moderate amounts of ABA helps maintain the normal activity of plant cells; however, when ABA is deficient in plants, their cellular activity may be reduced, resulting in increased sensitivity to adverse stress. For example, under high salt stress, it can activate ABA signaling, which promotes the accumulation of DELLA proteins, inhibits plant growth, and enhances resistance [62].

In the present study, all the *CsGRAS* genes were unevenly distributed on nine chromosomes, with most of the genes located on chromosome 5 and some genes from segmentally duplicated genes, which suggests that the expansion of the gene family is also largely dependent on the production of segmentally duplicated genes. In addition, this study further compared the covariance between sweet orange and two dicotyledonous plants, indicating that, among the two dicotyledonous plants, the covariance between sweet orange and its close relative, apple, was greater than that of the model species, *A. thaliana*.

When plants face adverse stress, they initiate a series of specific life activities as a response mechanism to ensure the stability of the intracellular environment and the maintenance of regular functions. During this process, the expression of some genes changes significantly, and these significantly different genes are likely to play a key role in the stress response pathway of plants [63]. In this study, we analyzed the expression patterns of *CsGRAS* genes under salt stress conditions and identified seven genes that were significantly differentially expressed. Among these, five downregulated genes (*CsGRAS19*, *CsGRAS20*, *CsGRAS25*, *CsGRAS1*, and *CsGRAS5*) belong to the HAM (HAIRY MERISTEM) subfamily within the GRAS transcription factor family. The HAM subfamily is evolutionarily conserved and plays crucial roles in plant development, most notably in maintaining the undifferentiated state of the shoot apical meristem (SAM) through its specific expression patterns and regulatory mechanisms [64]. The SAM is responsible for generating new cells and tissues for continuous plant growth and is the most actively proliferating region of the plant. While HAM genes have also been implicated in root development [64], their primary function centers on SAM regulation. These phenotypic alterations are consistent with established markers of ionic toxicity and oxidative damage under salt stress conditions [65]. The inclusion of *CsGRAS34*, *CsGRAS26*, *CsGRAS43*, *CsGRAS11*, and *CsGRAS35* within the *CsGRAS35* subfamily—despite their primary classification in other subfamilies—suggests shared structural motifs, functional convergences, or conserved evolutionary origins that transcend conventional subfamily boundaries. This observation challenges rigid subfamily delineations and implies that divergent evolution, gene duplication events, or domain shuffling may have generated paralogs with overlapping characteristics. Such complexity likely reflects recurrent adaptive processes within the *GRAS* gene family, where subfunctionalization or neofunctionalization following gene duplication could drive sequence diversification while preserving core regulatory roles. The recurrent presence of *CsGRAS35* orthologs across subfamilies further underscores the dynamic evolutionary trajectory of *GRAS* genes. This pattern may arise from lineage-specific duplication, unequal recombination, or selective retention of ancestral genes with pleiotropic functions. These mechanisms collectively contribute to functional plasticity within the family, enabling nuanced regulatory responses to environmental stimuli. Notably, *CsGRAS15* represents a distinct functional outlier. Its absence from GO annotations suggests uncharacterized molecular roles or taxon-specific functions not yet captured by existing databases. In contrast, KEGG pathway analysis associates *CsGRAS15* and *CsGRAS27* with gibberellin (GA) signaling cascades, where both encode DELLA proteins—central repressors of GA responses. Crucially, DELLA proteins act as master regulators of GA-mediated growth modulation as follows: their degradation derepresses GA signaling, while their stabilization (e.g., under stress) suppresses it. We propose that CsGRAS15 and *CsGRAS27* derived DELLA proteins orchestrate salt stress adaptation by directly tuning GA activity levels, thereby modulating growth–defense trade-offs.

## 5. Conclusions

This study identified 43 CsGRAS transcription factors in the sweet orange genome, phylogenetically classified into ten subfamilies. The transcriptome analysis revealed 42 salt-responsive *CsGRAS* genes (19 upregulated and 23 downregulated), with 7 exhibiting significant differential expression. qRT-PCR validation confirmed these expression patterns. The integrated analysis (phylogeny, homology, and structure) linked *CsGRAS* genes to known arabidopsis salt-tolerance pathways. Notably, KEGG enrichment implicated DELLA subfamily genes in gibberellic acid (GA) signaling. Two significantly differentially expressed DELLA genes, *CsGRAS15* and *CsGRAS27*, encode DELLA proteins—negative regulators of GA response that modulate growth under salt stress. *CsGRAS15* exhibits unique characteristics (KEGG enrichment without GO annotation), suggesting pathway-specific functionality. Both DELLA genes possess enriched light-responsive cis-elements, aligning with reports linking light-induced DELLA accumulation to salt tolerance. This comprehensive analysis provides a foundation for understanding *CsGRAS* function and enables targeted breeding of salt-tolerant citrus cultivars through the manipulation of key regulators like *CsGRAS15/27*.

## Data Availability

All the data generated or analyzed during this study are included in this published article. The C. sinensis genome (v3.0) and annotation files of sweet orange are openly available in the CPBD: Citrus Pangenome to Breeding Database (http://citrus.hzau.edu.cn/seqFetch/query.php (accessed on 6 March 2024)). RNA-Seq data under salt stress conditions can be found under accession numbers GSE248845, GSM7921250, GSM7921251, GSM7921252, GSM7921253, and GSM7921255. The RNA-Seq data are publicly available at the National Center for Biotechnology Information. The other data presented in this study are available in the Supplementary Materials.

## Supplementary Materials

The following supporting information can be downloaded at: https://www.mdpi.com/article/10.3390/biom15070946/s1, Table S1: Primer sequences used for qRT-PCR; Table S2: Gene renaming and the subfamily to which it belongs; Table S3: Analysis of physicochemical properties of CsGRAS TFs; Table S4: GO enrichment analysis of sweet orange under salt stress; Table S5: Analysis of KEGG enrichment in sweet orange under salt stress; Table S6: FPKM values of CsGRAS genes; Table S7: Raw CT values of qRT-PCR data for the GRAS gene; Figure S1: Heat map of CsGRAS sequence similarity matrix; Figure S2: Multiple sequence comparison of sweet orange GRAS protein.
