# Supplementary material for: Genome-Wide Identification of the GRAS Transcription Factor Family in Sweet Orange and the Regulation of Salt Stress-Enhanced Plant Salt Tolerance in Sweet Orange by CsGRAS15 and CsGRAS27
Source: Biomolecules. 2025 Jun 29;15(7):946. doi: 10.3390/biom15070946 (PMC12292939; doi:10.3390/biom15070946)
Supplement: Supplementary file 1 [file biomolecules-15-00946-s001.zip › biomolecules-3665573-supplementary.pdf]

Table S1. Primer sequences used for qRT-PCR.

**Gene**		**sequence（5'-3'）**
Actin	F	GGTCGTGATCTAACAGACGCCTT
	R	ATCAAGGGCAACATACGCAAGT
CsGRAS15	F	CAGTGGCCGGCTCTTTGA
	R	TCCGAAAGCTCAACGCGT
CsGRAS27	F	CACAGTTACGGTGGCGGT
	R	TAACTCGTCCATGCCGCC
CsGRAS35	F	TCCTTCAGATGCACCGCC
	R	CTAAGGTGCGCCGGTTCA
CsGRAS38	F	CAGCAAGCCCGTTCAGGA
	R	TTGGCGCTGGATGAAGCA

Table S2. Gene renaming and the subfamily to which it belongs.

gene id	rename	subfamily	species
AT1G07520.3	AtSCL31	LISCL	Arabidopsis thaliana
AT1G07530.1	AtSCL14	LISCL	Arabidopsis thaliana
AT1G14920.1	AtGAI	DELLA	Arabidopsis thaliana
AT1G21450.1	AtSCL1	PAT1	Arabidopsis thaliana
AT1G50420.1	AtSCL3	SCL3	Arabidopsis thaliana
AT1G50600.2	AtSCL5	PAT1	Arabidopsis thaliana
AT1G55580.1	AtLAS	SCL4/7	Arabidopsis thaliana
AT1G63100.2	AtSCL28	DLT	Arabidopsis thaliana
AT1G66350.1	AtRGL1	DELLA	Arabidopsis thaliana
AT2G01570.1	AtRGA	DELLA	Arabidopsis thaliana
AT2G04890.1	AtSCL21	PAT1	Arabidopsis thaliana
AT2G29060.1	AtSCL33	LISCL	Arabidopsis thaliana
AT2G37650.1	AtSCL9	LISCL	Arabidopsis thaliana
AT2G45160.1	AtHAM1	HAM	Arabidopsis thaliana
AT3G03450.1	AtRGL2	DELLA	Arabidopsis

			thaliana
AT3G13840.1	AtSCL29	SHR	Arabidopsis thaliana
AT3G46600.1	AtSCL30	LISCL	Arabidopsis thaliana
AT3G49950.1	AtSCL32	SHR	Arabidopsis thaliana
AT3G50650.1	AtSCL7	SCL4/7	Arabidopsis thaliana
AT3G54220.1	AtSCR	SCR	Arabidopsis thaliana
AT3G60630.1	AtHAM2	HAM	Arabidopsis thaliana
AT4G00150.1	AtHAM3	HAM	Arabidopsis thaliana
AT4G08250.1	AtSCL26	SCL4/7	Arabidopsis thaliana
AT4G17230.1	AtSCL13	PAT1	Arabidopsis thaliana
AT4G36710.1	AtSCL15	HAM	Arabidopsis thaliana
AT4G37650.1	AtSHR	SHR	Arabidopsis thaliana
AT5G17490.1	AtRGL3	DELLA	Arabidopsis thaliana
AT5G41920.1	AtSCL23	SCR	Arabidopsis thaliana
AT5G48150.1	AtPAT1	PAT1	Arabidopsis thaliana
AT5G52510.1	AtSCL8	PAT1	Arabidopsis thaliana
AT5G59450.1	AtSCL11	LISCL	Arabidopsis thaliana
AT5G66770.1	AtSCL4	SCL4/7	Arabidopsis thaliana
Cs_ont_1g002080.1	CsGRAS1	HAM	Citrus sinensis
Cs_ont_1g019320.1	CsGRAS2	SHR	Citrus sinensis
Cs_ont_1g023430.1	CsGRAS3	SHR	Citrus sinensis
Cs_ont_1g023860.1	CsGRAS4	SHR	Citrus sinensis
Cs_ont_1g026300.1	CsGRAS5	HAM	Citrus sinensis
Cs_ont_1g026590.1	CsGRAS6	SCL4/7	Citrus sinensis
Cs_ont_2g000710.1	CsGRAS7	PAT1	Citrus sinensis
Cs_ont_2g013070.1	CsGRAS8	DELLA	Citrus sinensis
Cs_ont_2g017480.1	CsGRAS9	PAT1	Citrus sinensis

Cs_ont_2g019960.1	CsGRAS10	SHR	Citrus sinensis
Cs_ont_2g024650.1	CsGRAS11	CsGRAS35	Citrus sinensis
Cs_ont_2g031350.1	CsGRAS12	SCL3	Citrus sinensis
Cs_ont_3g008170.1	CsGRAS13	PAT1	Citrus sinensis
Cs_ont_3g009590.1	CsGRAS14	SCL4/7	Citrus sinensis
Cs_ont_4g006820.1	CsGRAS15	DELLA	Citrus sinensis
Cs_ont_4g016660.1	CsGRAS16	PAT1	Citrus sinensis
Cs_ont_4g020090.1	CsGRAS17	PAT1	Citrus sinensis
Cs_ont_4g021880.1	CsGRAS18	SCL4/7	Citrus sinensis
Cs_ont_5g001380.1	CsGRAS19	HAM	Citrus sinensis
Cs_ont_5g001400.1	CsGRAS20	HAM	Citrus sinensis
Cs_ont_5g006980.1	CsGRAS21	SCL3	Citrus sinensis
Cs_ont_5g012210.1	CsGRAS22	SCR	Citrus sinensis
Cs_ont_5g018360.1	CsGRAS23	SHR	Citrus sinensis
Cs_ont_5g040500.1	CsGRAS24	DLT	Citrus sinensis
Cs_ont_5g042080.1	CsGRAS25	HAM	Citrus sinensis
Cs_ont_5g044300.1	CsGRAS26	CsGRAS35	Citrus sinensis
Cs_ont_6g005150.1	CsGRAS27	DELLA	Citrus sinensis
Cs_ont_6g011730.1	CsGRAS28	SCR	Citrus sinensis
Cs_ont_6g012290.1	CsGRAS29	SCR	Citrus sinensis
Cs_ont_6g014130.1	CsGRAS30	SCL4/7	Citrus sinensis
Cs_ont_6g014140.1	CsGRAS31	SCL4/7	Citrus sinensis
Cs_ont_6g017210.1	CsGRAS32	LISCL	Citrus sinensis
Cs_ont_7g000170.2	CsGRAS33	LISCL	Citrus sinensis
Cs_ont_7g022990.1	CsGRAS34	CsGRAS35	Citrus sinensis
Cs_ont_7g027840.1	CsGRAS35	SCL3	Citrus sinensis
Cs_ont_8g002330.1	CsGRAS36	LISCL	Citrus sinensis
Cs_ont_8g002350.1	CsGRAS37	LISCL	Citrus sinensis
Cs_ont_8g002360.1	CsGRAS38	LISCL	Citrus sinensis
Cs_ont_8g002370.1	CsGRAS39	LISCL	Citrus sinensis
Cs_ont_9g001540.1	CsGRAS40	SCL3	Citrus sinensis
Cs_ont_9g001580.1	CsGRAS41	SCL3	Citrus sinensis
Cs_ont_9g026300.1	CsGRAS42	PAT1	Citrus sinensis
Cs_ont_9g028270.1	CsGRAS43	CsGRAS35	Citrus sinensis

Table S3. Analysis of physicochemical properties of CsGRAS TFs.

sequence ID	gene name	Number of amino acids (aa)	Molecular weight (kDa)	Theoretical pI	Instability index	Aliphatic index	Subcellular Localization
Cs_ont_1g002080	CsGRAS1	609	67.24	5.99	50.29	85.39	Nucleus
Cs_ont_1g019320	CsGRAS2	561	62.13	5.58	54.29	72.00	Nucleus

Cs_ont_1g023430	CsGRAS3	490	55.27	5.17	47.99	70.61	Nucleus
Cs_ont_1g023860	CsGRAS4	459	51.44	5.80	42.01	89.93	Nucleus
Cs_ont_1g026300	CsGRAS5	578	64.61	6.48	53.1	89.10	Nucleus
Cs_ont_1g026590	CsGRAS6	622	68.64	5.21	52.36	76.75	Nucleus
Cs_ont_2g000710	CsGRAS7	547	61.35	5.59	54.47	77.35	Nucleus
Cs_ont_2g013070	CsGRAS8	594	65.01	5.07	50.32	78.10	Nucleus
Cs_ont_2g017480	CsGRAS9	536	59.84	5.69	56.56	82.46	Nucleus
Cs_ont_2g019960	CsGRAS10	449	49.86	5.63	48.72	91.02	Nucleus
Cs_ont_2g024650	CsGRAS11	424	47.12	6.32	48.78	79.41	Nucleus
Cs_ont_2g031350	CsGRAS12	715	77.82	6.18	39.41	99.64	Nucleus
Cs_ont_3g008170	CsGRAS13	587	55.57	5.05	51.83	77.61	Nucleus
Cs_ont_3g009590	CsGRAS14	434	47.48	5.27	41.27	79.17	Nucleus
Cs_ont_4g006820	CsGRAS15	531	58.18	5.40	52.68	89.47	Nucleus
Cs_ont_4g016660	CsGRAS16	580	63.96	6.05	54.03	83.43	Nucleus
Cs_ont_4g020090	CsGRAS17	545	60.91	5.53	55.19	80.24	Nucleus
Cs_ont_4g021880	CsGRAS18	457	51.79	6.68	54.89	96.52	Nucleus
Cs_ont_5g001380	CsGRAS19	706	78.27	5.64	59.91	83.17	Nucleus
Cs_ont_5g001400	CsGRAS20	702	77.06	5.48	54.49	88.40	Nucleus
Cs_ont_5g006980	CsGRAS21	606	68.35	5.90	42.9	88.50	Nucleus
Cs_ont_5g012210	CsGRAS22	519	58.44	6.10	48.28	80.64	Nucleus
Cs_ont_5g018360	CsGRAS23	460	51.81	5.28	42.59	80.33	Nucleus
Cs_ont_5g040500	CsGRAS24	667	74.00	5.83	51.37	77.18	Nucleus
Cs_ont_5g042080	CsGRAS25	706	77.88	6.14	50.92	81.88	Nucleus
Cs_ont_5g044300	CsGRAS26	514	57.20	6.84	52.24	89.96	Nucleus
Cs_ont_6g005150	CsGRAS27	572	63.14	5.06	47.93	80.37	Nucleus
Cs_ont_6g011730	CsGRAS28	441	48.41	5.16	59.61	96.92	Nucleus
Cs_ont_6g012290	CsGRAS29	824	90.03	6.06	58.15	80.21	Nucleus
Cs_ont_6g014130	CsGRAS30	502	57.14	4.80	43.07	41.49	Nucleus
Cs_ont_6g014140	CsGRAS31	588	66.77	6.06	51.53	78.49	Nucleus
Cs_ont_6g017210	CsGRAS32	747	84.94	6.03	52.74	75.84	Nucleus
Cs_ont_7g000170	CsGRAS33	749	84.62	5.54	50.89	76.01	Nucleus
Cs_ont_7g022990	CsGRAS34	503	56.37	6.92	46.71	89.18	Nucleus
Cs_ont_7g027840	CsGRAS35	480	53.86	5.88	62.99	91.08	Nucleus
Cs_ont_8g002330	CsGRAS36	700	79.16	6.16	50.41	71.04	Nucleus
Cs_ont_8g002350	CsGRAS37	685	77.22	6.24	46.55	73.20	Nucleus
Cs_ont_8g002360	CsGRAS38	632	71.85	6.06	43.3	71.31	Nucleus
Cs_ont_8g002370	CsGRAS39	800	90.00	5.24	43.27	70.20	Nucleus
Cs_ont_9g001540	CsGRAS40	600	68.55	5.07	47.27	84.65	Nucleus
Cs_ont_9g001580	CsGRAS41	430	48.53	6.07	45.09	93.65	Nucleus
Cs_ont_9g026300	CsGRAS42	659	72.98	6.46	53.21	75.60	Nucleus
Cs_ont_9g028270	CsGRAS43	540	60.83	5.23	47.62	87.09	Nucleus

Table S4. GO enrichment analysis of sweet orange under salt stress

ID	classification	Pvalue	all	up_gene	down_gene
GO:0003674	Molecular Function	27	24	8	10
GO:0003676	Molecular Function	16	24	8	10
GO:0003677	Molecular Function	9	24	8	10
GO:0003700	Molecular Function	18	24	8	10
GO:0005488	Molecular Function	26	24	8	10
GO:0043565	Molecular Function	21	24	8	10
GO:0097159	Molecular Function	19	24	8	10
GO:0140110	Molecular Function	35	24	8	10
GO:1901363	Molecular Function	6	24	8	10
GO:0001067	Molecular Function	22	1	0	1
GO:0044212	Molecular Function	25	1	0	1
GO:0006355	Biological Process	7	24	8	10
GO:0008150	Biological Process	31	24	8	10
GO:0009889	Biological Process	19	24	8	10
GO:0009987	Biological Process	26	10	4	6
GO:0010468	Biological Process	33	24	8	13
GO:0010556	Biological Process	36	24	8	13
GO:0019219	Biological Process	15	24	8	13
GO:0019222	Biological Process	17	24	8	13
GO:0030154	Biological Process	10	24	8	13
GO:0031323	Biological	39	24	8	13

	Process				
GO:0031326	Biological Process	20	24	8	13
GO:2001141	Biological Process	26	24	8	13
GO:0048518	Biological Process	28	3	0	3
GO:0046677	Biological Process	14	2	1	1
GO:0005575	Cellular Component	32	24	8	13
GO:0005622	Cellular Component	12	24	8	13
GO:0005623	Cellular Component	23	24	8	13
GO:0005634	Cellular Component	29	24	8	13
GO:0043226	Cellular Component	35	24	8	13
GO:0043227	Cellular Component	17	24	8	13
GO:0043229	Cellular Component	26	24	8	13
GO:0043231	Cellular Component	22	24	8	13
GO:0044424	Cellular Component	19	24	8	13
GO:0044464	Cellular Component	36	20	6	9
GO:0005737	Cellular Component	24	5	2	3

Table S5. Analysis of KEGG enrichment in sweet orange under salt stress

ID	Count	Description
ko04075	5	Plant hormone signal transduction
ko00240	1	Pyrimidine metabolism

Table S6. FPKM values of *CsGRAS* genes

Name	CK1_fpkm	CK2_fpkm	CK3_fpkm	T1_fpkm	T2_fpkm	T3_fpkm
CsGRAS1	2.64	4.69	3.42	4.64	5.20	4.63
CsGRAS2	0.743	3.5453	1.8631	2.296	2.9691	3.2814
CsGRAS3	2.9499	3.22	1.8738	1.50729	1.33973	1.549

CsGRAS4	0.024	0.113	0	0.19	0	0.0217
CsGRAS5	2.7910	3.1050	3.93728	2.88498	2.1843	3.50512
CsGRAS6	3.32794	4.59	4.628	6.3933	2.6806	6.3688
CsGRAS7	171.673	284.31	205.37	157.34	163.449	157.84
CsGRAS8	11.735	16.368	11.960	17.837	6.7713	14.7551
CsGRAS9	4.5604	11.4229	4.983	9.1099	7.4019	8.20394
CsGRAS10	0.292747	0.03138	0.03261	0.2429	0.4205	0.4308
CsGRAS11	0	0	0.05474	0.0453	0.044	0.0401
CsGRAS12	23.862	32.392	27.883	25.4736	22.52	26.018
CsGRAS13	71.51	51.392	71.202	57.048	57.9473	66.726
CsGRAS14	0.4364	0.6690	0.802	0.6198	0.56	0.6673
CsGRAS15	2.355	3.4289	1.9734	2.5842	0.427	1.253
CsGRAS16	633.89	404.303	895.013	240.95	102.6542	119.141
CsGRAS17	85.741	63.758	111.105	62.471	67.614	61.4848
CsGRAS18	0	0	0.041	0	0.0336	0
CsGRAS19	4.054	6.523	5.726	5.012	6.417	5.7723
CsGRAS20	2.5924	4.108	2.54	5.287	4.373	7.335
CsGRAS21	0	0	0	0	0.0286	0
CsGRAS22	0.185	0	0.3783	0	0	0.0277
CsGRAS23	0.5709	0.6059	0.6648	0.608	0.3101	0.4878
CsGRAS24	0.9789	1.1956	0.235	0.195	0.08	0.2201
CsGRAS25	3.893	8.3279	5.298	5.9387	5.9933	7.1732
CsGRAS26	0	0	0	0.0747	0	0
CsGRAS27	5.069	9.6112	5.9288	13.74	15.019	19.727
CsGRAS28	15.438	9.334	14.38	7.3094	6.8494	9.8309
CsGRAS29	1.2719	1.4624	1.6564	2.226	0.5181	0.9438
CsGRAS30	9.6741	10.672	4.85773	8.101	21.975	13.1646
CsGRAS32	10.3888	20.453	11.323	16.2494	18.3006	17.3834
CsGRAS33	7.0618	9.613	4.105	14.2	14.604	17.563
CsGRAS34	0	0	0	0.038	0.037	0.1355
CsGRAS35	1.218	2.088	0.723	4.5783	2.27	2.6033
CsGRAS36	46.558	46.39	58.65	33.86	31.8	31.13
CsGRAS37	102.50	87.2319	80.049	74.48	78.57	80.49
CsGRAS38	11.609	7.0804	11.237	4.397	2.14	6.386
CsGRAS39	143.51	121.925	122.75	102.49	105.33	95.012
CsGRAS40	1.4909	2.23	0.764	1.32	0.5622	1.097
CsGRAS41	0.0733	0	0.17979	0.186	0	0.0659
CsGRAS42	13.184	24.5978	15.515	9.35	32.32	15.73
CsGRAS43	0.2391	0.6849	0.67	0.5199	0.20245	1.106

Table S7. Raw CT values of qRT-PCR data for the *GRAS* gene.

ID		CK	T
Actin	CT1	22.28032134	22.50629516
	CT2	21.98854764	21.64239134

	CT3	22.64107692	22.32987703
	CT1	22.3033153	21.52253307
CsGRAS15	CT2	20.3795624	20.73850206
	CT3	20.83347809	21.00513776
	CT1	22.51469777	21.86152389
CsGRAS27	CT2	22.69619046	21.42459788
	CT3	22.52515377	21.31554334
	CT1	21.13472576	22.47130283
CsGRAS35	CT2	21.07620648	22.05431922
	CT3	21.73104391	22.8219684
	CT1	21.34727165	22.22172594
CsGRAS38	CT2	21.02264451	21.38636139
	CT3	20.68566842	22.03005722

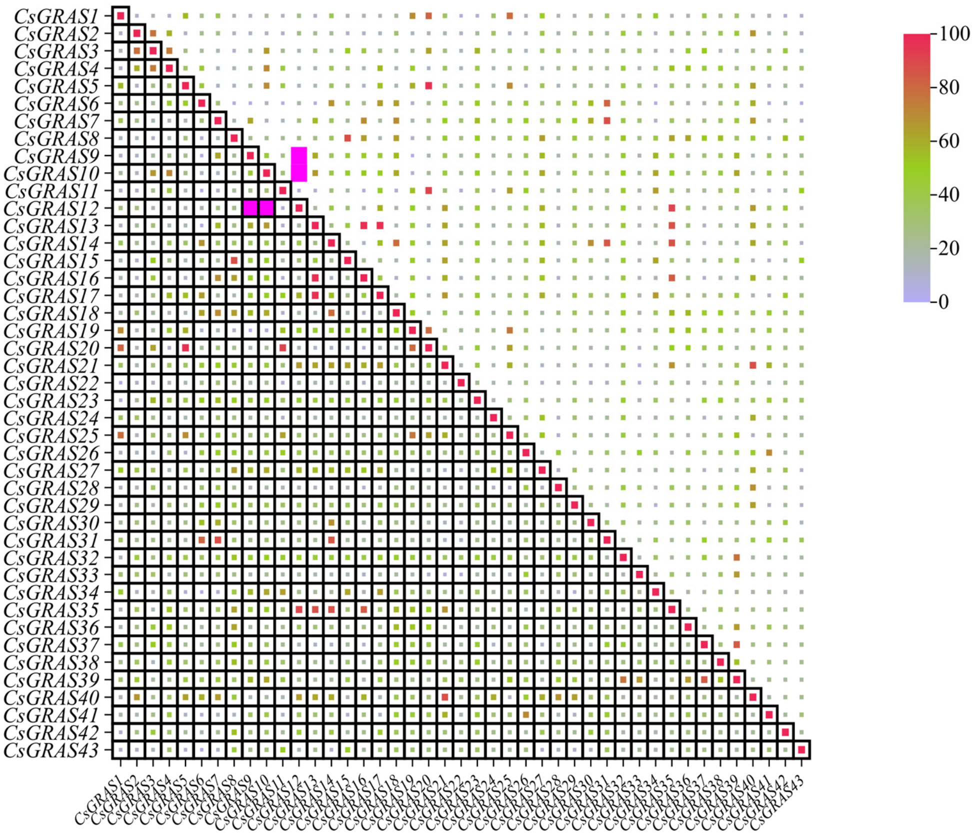

Figure S1. Heatmap for the sequence similarity analysis heatmap of the sequence similarity matrix of CsGRASs generated via TBtools software. The color indicates the percentage of sequence similarity; the redder the color, the higher the similarity, and vice versa, the lower the similarity; the color scale value is shown in the upper right.

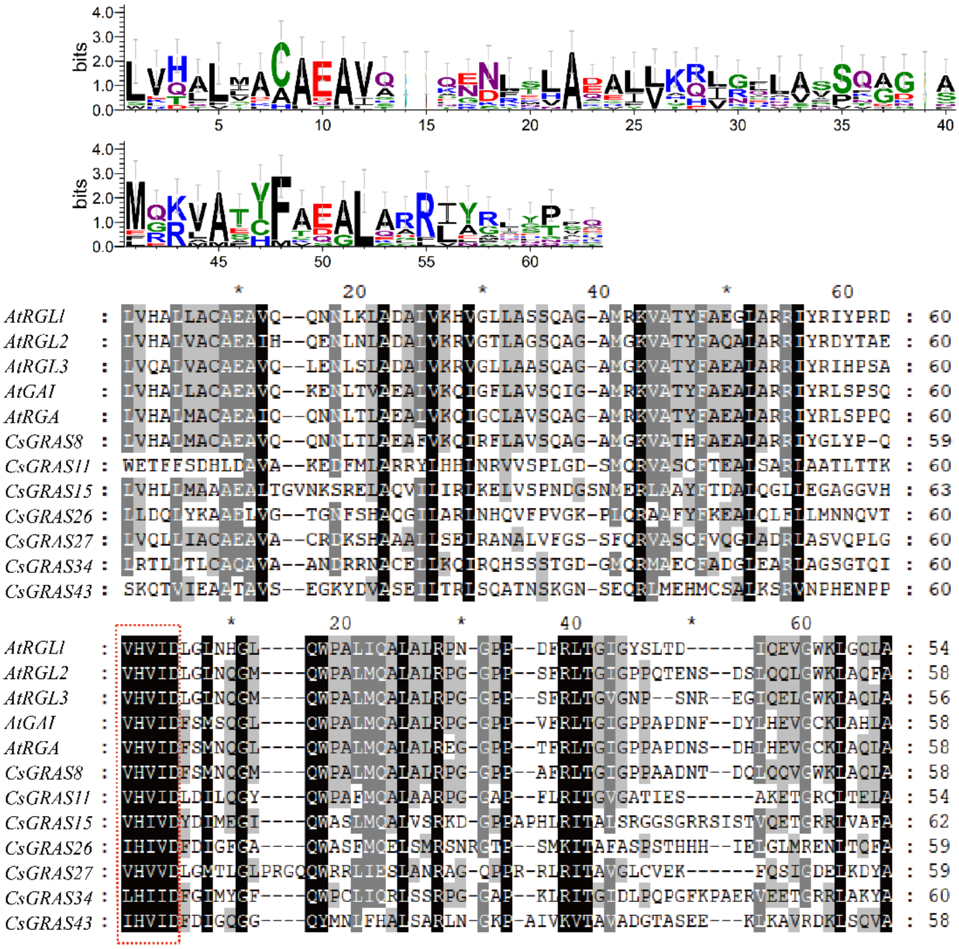

Figure S2. Multiple sequence comparison of sweet orange GRAS protein
